# Enhancing supermarket robot interaction: an equitable multi-level LLM conversational interface for handling diverse customer intents

**DOI:** 10.3389/frobt.2025.1576348

**Published:** 2025-04-29

**Authors:** Chandran Nandkumar, Luka Peternel

**Affiliations:** Department of Cognitive Robotics, Delft University of Technology, Delft, Netherlands

**Keywords:** voice interface, robotics, large language models, speech recognition, questionnaires

## Abstract

This paper presents the design and evaluation of a comprehensive system to develop voice-based interfaces to support users in supermarkets. These interfaces enable shoppers to convey their needs through both generic and specific queries. Although customisable state-of-the-art systems like GPTs from OpenAI are easily accessible and adaptable, featuring low-code deployment with options for functional integration, they still face challenges such as increased response times and limitations in strategic control for tailored use cases and cost optimization. Motivated by the goal of crafting equitable and efficient conversational agents with a touch of personalisation, this study advances on two fronts: 1) a comparative analysis of four popular off-the-shelf speech recognition technologies to identify the most accurate model for different genders (male/female) and languages (English/Dutch) and 2) the development and evaluation of a novel multi-LLM supermarket chatbot framework, comparing its performance with a specialized GPT model powered by the GPT-4 Turbo, using the Artificial Social Agent Questionnaire (ASAQ) and qualitative participant feedback. Our findings reveal that OpenAI’s Whisper leads in speech recognition accuracy between genders and languages and that our proposed multi-LLM chatbot architecture, which outperformed the benchmarked GPT model in performance, user satisfaction, user-agent partnership, and self-image enhancement, achieved statistical significance in these four key areas out of the 13 evaluated aspects that all showed improvements. The paper concludes with a simple method for supermarket robot navigation by mapping the final chatbot response to the correct shelf numbers to which the robot can plan sequential visits. Later, this enables the effective use of low-level perception, motion planning, and control capabilities for product retrieval and collection. We hope that this work encourages more efforts to use multiple specialized smaller models instead of always relying on a single powerful model.

## 1 Introduction

In recent times, the presence of robots in our daily lives has increased drastically and they are now capable of working side-by-side with humans to achieve a given objective. Bloss (2016) explains how these robots improve efficiency, reduce training times for operators, and provide better safety than their autonomous robot counterparts. Since collaborative robots are a vast and growing field in robotics (Goldberg, 2019), multiple works address the need for different approaches to provide efficient, immersive and aware control. Villani et al. (2018) make a strong argument for the need for intuitive user interfaces, which help reduce operation time and operator errors whilst maintaining situational awareness and engagement.

There are multiple options available to interact with collaborative robots. To furnish some examples (Anjum et al., 2023; Kragic et al., 2018; Takarics et al., 2008), show different implementations of robot collaboration using vision for a variety of applications like pick-and-place to welding; (García et al., 2022; Senft et al., 2021); presents the implementation of Augmented reality for human-robot collaborative surface treatment and task-level authoring, respectively, whilst (Liu et al., 2019; Solanes et al., 2022) present the use case of Virtual Reality for the control of robotic manipulators and mobile robots. There are various other means of controlling a robot, like eye tracking, pose determination, haptics, facial expressions and more. Furthermore, it is also possible to use multiple such interfaces simultaneously to get more precise and accurate results, as seen in Shaif et al. (2023) and Bai et al. (2023).

One area of particular interest for this work is voice. Voice-based interaction being the default means of communication between humans, holds great promise in being applied to robots. It can allow for more authentic conversations and communication of user intent as opposed to other means and also benefits from being hands-free, allowing human users to manipulate other objects and their environment. However, due to previous limitations in both speech recognition and language processing, most of the implementations of voice-based command of robots were rather primitive as they allowed only a few distinct and restricted voice commands as seen in (Norberto Pires, 2005; Rogowski, 2012) preventing robots from achieving natural language voice interaction with human users.

The first area of focus is ensuring our system is equitable to relevant user demographics in Dutch supermarkets, and is ensured by the robustness of the Voice-to-text conversion, facilitated via Automatic Speech Recognition (ASR) systems. ASR has been an important area of research in the domain of natural language processing and computational linguistics, with the promise of playing a crucial role in bettering human-machine interaction (Malik et al., 2021). ASR systems are built to transcribe a given intelligent auditory signal into its linguistic textual counterpart and differ from speech understanding–ASR by itself cannot operate or extract information from the signal (Levis and Suvorov, 2012). Over the past few years, it has become very easy for people to transcribe their voice using many free and low-cost ASR systems released by extremely popular players such as Google, OpenAI, Microsoft, Meta and more. While each of these models offers its own advantages and disadvantages, it is crucial to measure how robust these systems are to the voices of different users and provide recommendations on the ideal ASR system for robot interaction. Since the text generated by the ASR system will be subsequently used by a Large Language Model the system selected must be as fair and inclusive as possible for the target users.

The second area involves bringing together the two areas of interest from above and building a novel multi-LLM hierarchical conversational agent capable of responding to all kinds of queries and modifying the final bill of the user. This system is evaluated against the present state-of-the-art GPT created with the same data and information provided to our approach. The goal here is to compare how our approach performs against the state-of-the-art on the Artificial Social Agent Questionnaire (ASAQ), a popular questionnaire to evaluate chatbots.

### 1.1 Problem statement

Despite voice-based interfaces having significant advantages for human-robot interaction, there are a number of concerns that must be addressed if one intends to build an equitable and highly usable conversational agent.

Firstly, the selection of the right speech recognition system based on the target application and demographic is essential to ensure that the conversational agent can handle variations in language, gender and accent robustly and uniformly for all speakers. This however has not been the case for most popular speech recognition systems that have been trained largely on highly biased datasets, making them considerably erratic with certain demographics. In Rajan et al. (2022), an approach on automated fairness testing of speech recognition systems found that “non-native English, female and Nigerian English speakers generate 109%, 528.5% and 156.9% more errors, on average, than native English, male and UK Midlands speakers, respectively.” Hutiri and Ding (2022) state that female and non-US nationalities experience significant performance degradation when using automated speech recognition systems. Koenecke et al. (2020) found that five popular ASR systems by Apple, Amazon, Google, Microsoft and IBM exhibited substantial racial disparities, with an average word error rate (WER) of 0.35 for black speakers compared with 0.19 for white speakers.

Secondly, the deployed chatbots must be capable of performing the task as efficiently and usefully as possible. Given the variability in types of requests in terms of complexity and degree of language processing required, a supermarket chatbot must be able to respond to straightforward queries such as asking a specific item availability, position and price to significantly more open-ended and broader queries regarding high-level intents such as recommendations for a specific dinner or items required for a party. Chatbots built by LLMs are also prone to significant hallucinations and mistakes, which influence the degree of trust users can place in these systems Ma et al. (2023). Furthermore, the latency of such systems is often extremely high, affecting their degree of usability. Thus, a highly robust and adaptable system that is capable of understanding the user’s priorities and needs must be developed to ensure it can be beneficial to supermarket customers.

### 1.2 Research questions and objectives

The primary aim of this paper is to answer the following research question -

How can voice-based supermarket robot interfaces be designed to enhance parity across gender and language, while maintaining high usability, as evidenced by evaluations of ASR robustness and multi-LLM effectiveness compared to the state-of-the-art?

This research question can thus be broken down into two sub-research questions:

1. Which off-the-shelf speech recognition system emerges as the most robust to variation in speaker gender(male/female) and language(English/Dutch)?
2. How does our novel multi-LLM conversational agent fare on the Artificial Social Agent Questionnaire (ASAQ) against the state-of-the-art GPT4 Turbo-powered GPT agent in a human factors experiment?

The objective of this paper is to present a novel approach to addressing the width and depth of user queries in a supermarket by providing the best possible results. The final chatbot can also be integrated with a mobile base robot to enable the robot to navigate to the correct shelves before the low-level control of a manipulator, perception and object retrieval can be incorporated to get the object from the shelf.

The paper is structured as follows. Section 2 will cover all the relevant terms, base technologies and previous work covered in the field. Section 3 will go in-depth with respect to the first component–evaluation of off-the-shelf speech recognition, including experiment setup and evaluation. Section 4 proposes our very own approach and how it has been built, along with the experiment setup to compare it against the state-of-the-art. Section 5 involves the results of all experiments. Section 6 includes the discussion and future work recommendations. Lastly, Section 7 is the conclusion.

## 2 Methods - speech recognition

Before we dive into the speech recognition experiment, we introduce the 4 models, relevant metrics and underlying technologies to set the stage for our contributions. The Automatic Speech Recognition (ASR) system is the first component of voice-based interfaces, as it enables the conversion of speech into text, which can then be processed for further downstream tasks. The selection of the right ASR system is essential as it is the most important component in the conversational agent for ensuring parity and equitability for different customers. Speech recognition systems that are biased tend to perform worse for certain demographics making the usage of such systems difficult for these groups.

### 2.1 Different ASR systems

Whilst there are many different speech recognition systems offered by multiple providers, this thesis will explore 4 prominent ones. They are selected based on their relevance, ability to transcribe Dutch and general popularity. We pick 2 open-source systems (Vosk and Whisper by OpenAI) and 2 closed-source systems (Google Cloud Speech-to-text and Microsoft Azure speech-to-text). This provides a good balance in terms of the different capabilities of the system, such as online vs offline use, free vs paid, the ability to fine-tune locally for specific use cases, data privacy and security and so on.

#### 2.1.1 Vosk

Vosk is an open-source speech recognition toolkit that has made its mark in offline voice recognition and remains relevant despite being older than its counterparts Alpha Cephei (2023). Unlike many cloud-based solutions, Vosk operates entirely on the device, ensuring data privacy and enabling voice recognition even in the absence of an internet connection. Underlying Vosk’s capabilities is the Kaldi engine whose origin dates back to 2009. Developed in C++, Kaldi is an open-source speech recognition toolkit that can be effortlessly deployed across multiple operating systems. While initial versions of Kaldi primarily supported the English language, over time it has grown to support over 20 languages, including Dutch.

Vosk itself, also written in C++, offers bindings for a plethora of programming languages, including Python, Java, and Node.js, making it remarkably versatile in its applications. From smaller devices like the Raspberry Pi to extensive server configurations, Vosk can be tailored to a wide array of scenarios. It offers pre-trained models for various languages, giving developers a significant head-start to implement them for speech recognition applications. Additionally, the toolkit supports custom model training, allowing for its deployment in niche contexts or for less common languages. Vosk has found its utility in diverse applications, such as voice assistants, transcription services, and voice-driven gaming. Its notable strengths encompass its adaptability, support of multiple languages, portability, offline functionality, and open-source support Andreev and Chuvilin (2021). Its limitations include higher resource consumption due to offline use, lower accuracy than more advanced proprietary models and reduced general functionality. Furthermore, Vosk has different models for different languages, requiring a language classifier to be present at the input to select the right model for transcription.

#### 2.1.2 Whisper

Whisper by OpenAI is a cutting-edge ASR system, remarkable for its training on an expansive 680,000 h of multi-task, multilingual data harvested from the web Radford et al. (2022). This enormous and diverse training foundation endows Whisper with notable robustness against various challenges, be it accents, technical jargon, or ambient noise. It is also capable of handling multiple languages and translation between them as well a feat made possible since about a third of the data is in other languages.

Whisper’s architecture is based on an end-to-end Encoder-Decoder Transformer model. Audio inputs are first segmented into 30-second blocks, which undergo preprocessing before being fed into the encoder. The decoder, in turn, is meticulously trained to predict corresponding text captions. One key standout feature of Whisper is the incorporation of specialized tokens that instruct the model to undertake varied tasks, from identifying languages and time-stamping phrases to multilingual transcription and translation into English.

While most ASR models lean on smaller, tightly coupled audio-text datasets or unsupervised audio pre-training, Whisper is trained on a broad and varied training set. Thus, although it might not outshine models on niche benchmarks like LibriSpeech, it is capable of unparalleled robustness across a myriad of diverse datasets, reducing Whisper’s error rate by half compared to other more specialized models.

#### 2.1.3 Google Cloud speech-to-text

Google Cloud Speech-to-Text API is an ASR system developed by Google Bohouta and Këpuska (2017). It is part of the larger suite of Google Cloud services and is designed to convert audio to text with high accuracy and efficiency. The API leverages Google’s advanced machine-learning models and is capable of recognizing over 125 languages and their variants. It can be used in real-time applications or to transcribe pre-recorded audio files.

One of the key merits of the Google Cloud Speech-to-Text API is its high accuracy, even in noisy environments and with different accents. It also provides real-time transcription, which is crucial for applications like voice assistants and real-time captioning. Additionally, the API offers features like speaker identification, enabling different speakers in a conversation to be recognised, and word-level confidence scores, which can be used to identify uncertain parts of the transcription. Previous work has also shown that Google has the lowest WER compared to Microsoft Azure Speech and CMU Sphinx, another popular albeit less effective open-source ASR system Bohouta and Këpuska (2017).

#### 2.1.4 Microsoft Azure Speech

Microsoft Azure Speech is a proprietary speech recognition and transcription service by Microsoft Bohouta and Këpuska (2017). Microsoft has continued to develop powerful speech APIs for many years and has released a series of increasingly powerful speech platforms. Microsoft has in the past used context-dependent deep neural network hidden Markov model (CD-DNN-HMM). These CD-DNN-HMM models were able to achieve substantially better results than a Context-Dependent Gaussian Mixture Model Hidden Markov model (CD-GMM-HMM). In 2016, Microsoft also announced they had achieved human parity in speech recognition as published in the paper ‘Achieving Human Parity in Conversational Speech Recognition by using various convolutional/LSTM acoustic model architectures, novel spatial smoothing methods, lattice-free MMI acoustic training, multiple recurrent neural network language modelling approaches, and systematic use of the system combination to even beat professional transcribers and set new benchmarks Xiong et al. (2017).

These 4 ASR systems discussed above are extremely popular and used by multiple services for different applications. They can also be integrated into systems via a simple API call, making them ideal for robot voice interfaces. Now that we have introduced the 4 models that will be evaluated, we focus our attention on the metric to evaluate their performance.

### 2.2 Metric for comparing ASR systems

To evaluate the different ASR systems we use the Word Error Rate, a popular and simple metric to assess the accuracy of transcription by comparing the output of the system with the ground truth.

Word Error Rate (WER) is a popular metric used to evaluate the performance of automatic speech recognition and machine translation systems Morris (2002). It measures the difference between the words in a reference transcription and the words in the system’s output in terms of substitutions, insertions, and deletions needed to make the two match. These are the three different errors which could be introduced in transcribing and the WER helps us understand the ratio of these errors over the total number of input words expressed as a percentage. So the smaller the WER, the better the speech recognition system is at transcribing the spoken text Morris (2002).

The equation for WER can thus be written as:

$$\text{WER}=\frac{I+D+S}{N}$$ (1)

Where:

• I is the number of insertions.
• D is the number of deletions.
• S is the number of substitutions.
• H is the number of words present in the reference text

The WER as described in Equation 1, however, suffers from some limitations, such as not being D/I symmetric, i.e., it gives more importance to insertions than deletions when both of them are equally disadvantageous. Furthermore, it is not bounded and thus can exceed 100%. Besides WER, two other key metrics are Match Error Rate (MER) and Word Information Lost (WIL), which all evaluate similar key aspects. For this study, we chose to use WER due to its widespread adoption and consistency in prior research comparing ASR systems. Additionally, in our case, the difference between WER and MER is negligible, as the models under evaluation demonstrate high performance, and MER is particularly useful when assessing lower-performing models. While WIL provides valuable insights, it introduces additional complexity, and using multiple metrics could make the evaluation less straightforward. WER effectively captures the key aspects of recognition errors—insertions, deletions, and substitutions, and remains the default method used by the speech recognition community to measure the performance of their systems due to its simplicity and ease of understanding. In light of this, to maintain consistency with previous literature, we proceed with using WER.

## 3 Evaluation of speech recognition systems

After discussing the models and the evaluation metric used to measure ASR performance, we proceed with the experiment setup. Given the limited research that has gone into task-specific speech transcription and evaluation of the Dutch language, we proceed to perform a human factors experiment of 40 participants to pick the system that offers the highest accuracy despite variations in speaker gender and spoken language.

### 3.1 Participants

The 40 participants were divided into the following 4 equal and exclusive groups:

1. Dutch Female (DF)
2. Dutch Male (DM)
3. English Female (EF)
4. English Male (EM)

The groups were made exclusive to remove the potential influence of bilingualism since all Dutch speakers were proficient in English but the converse was not true. The experiment was approved by the Human Research Ethics team at Delft University of Technology. Participants were recruited from common public areas around the campus and word of mouth. Efforts were made to ensure the participants were from different nationalities and had diverse accents to make the study sample representational of the typical customers who would visit a supermarket. Participants were asked to sign the informed consent form. Before participants were asked to speak the lines of the script, an informed consent form was shown to clearly explain the format of data collection and privacy (Supplementary Material SC).

### 3.2 Experiment design

All participants were asked to read from either a given English or Dutch script. The script was custom-generated based on commonly used words in the supermarket, including product names, locations and other pieces of information, including words that are sometimes difficult to pronounce. The script featured a conversation between a customer and a helpful assistant (Supplementary Material S2). The audio was recorded using a FiFine USB microphone K669B and using the open-source tool Audacity on a Linux system in the MP3 format. The ambient noise during recording was minimized. While we acknowledge that supermarkets typically have significant background noise, we argue that employing noise-canceling techniques along with microphones positioned closer to the speaker’s mouth—such as wearable headbands or mobile phones—can significantly reduce interference–further reflecting the real-world deployment of such technologies alongside supermarket robots. The same audio file was then converted into the WAV format due to it being the versatile format accepted by all systems and used by the 4 speech recognition systems using the speech recognition library in Python - Microsoft Azure speech-to-text (model base), Google Cloud speech-to-text (model V1 - default), OpenAI Whisper (model large-V2) and Vosk (English - vosk-model-en-us-0.22-lgraph, Dutch - vosk-model-small-nl-0.22). For Azure, Google Cloud and Vosk, the language the user was speaking had to be specified, while Whisper was capable of recognising the language from the audio file alone. After the transcriptions were created by the models, they were saved as txt files and pre-processing was done to convert everything to lowercase and remove punctuation marks both in the reference script and the transcriptions. This was done because Google Cloud speech-to-text returned its output in lower case and penalising the absence of punctuation marks was not deemed necessary since the transcribed speech would be fed to an LLM–a system robust enough to handle such omissions. Using the reference script and created transcriptions, 4 Word Error Rates were calculated–one for each system.

The dependent variable in the experiment was the Word Error Rate of the transcribed text against the original script. The two independent variables are the speech recognition system–a within-subjects variable since the audio file of all participants was fed to all four systems and the group the participant belonged to based on their gender and language of the script. Since the 4 groups (DM, DF, EM, EF) were mutually exclusive, the group variable is a between-subjects factor. Thus, for the statistical analysis of these results, we first confirm the normality of all 16 columns of data (4 models x 4 groups) using the Shapiro-Wilk test. When normality of data was confirmed, a two-way mixed ANOVA was conducted. The threshold for statistical significance was set to 0.05.

### 3.3 Results of the evaluation of speech recognition systems

**FIGURE 1:**
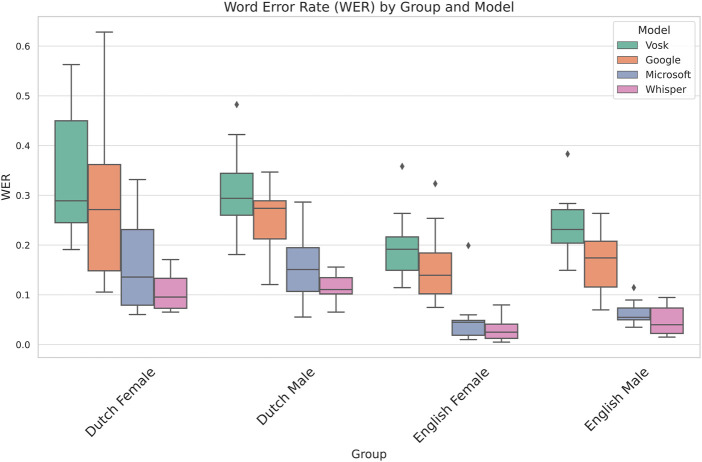
Comparison of different ASR systems across all models and groups. We observe that Dutch participants have a higher WER than their English counterparts.

We now outline the results obtained by comparing the Word Error Rate across the 4 speech recognition systems. Table 1 shows the results of the 2-way mixed ANOVA using model and group as the independent variables. We observe that the p-value of both group and gender is below 0.05 and thus statistically significant indicating that we have considerable differences in error rate on account of both the models and groups. Whilst this shows us that the group the participant belonged to affects the dependent variable, we do not get insights into which factor–language, gender or both is responsible for the variation in the performance of the models. To resolve this, we perform two more mixed ANOVA tests where we drop each of the factors and test for only one of the factors against the model. Tables 2, 3 take language and gender independently as between-subjects factors and maintain the model as between-subjects. Based on this, we observe that language has a significant influence on the WER while gender is not statistically significant. These insights are supported by the visualisation in Figure 1 where the difference between languages for participants of the same gender is lower than the differences between speakers of the same gender but different languages.

**TABLE 1:** ANOVA summary table for group versus model. We observe that the effect of the group has a statistically significant influence on the WER, but we do not have insights on which aspect of the group–gender, language or both–is responsible for it.

Source	SS	DF1	DF2	MS	F	p-unc	np2	eps
Group	0.373144	3	36	0.124381	7.350098	**5.772400e-04**	0.379848	-
Model	1.022612	3	108	0.340871	143.500414	**1.566141e-37**	0.799443	0.708692
Interaction	0.015847	9	108	0.001761	0.741261	0.6703523	0.058178	-

Bold p-vlues indicate statistically significant results.

**TABLE 2:** ANOVA Summary Table for Language versus Model. We observe that the effect of language has a statistically significant influence on the WER.

Source	SS	DF1	DF2	MS	F	p-unc	np2	eps
Language	0.361852	1	38	0.361852	22.160251	**3.296411e-05**	0.368354	-
Model	1.022612	3	114	0.340871	147.210089	**4.732748e-39**	0.794828	0.708692
Interaction	0.008419	3	114	0.002806	1.211918	3.087008e-01	0.030907	-

Bold p-vlues indicate statistically significant results.

**TABLE 3:** ANOVA summary table for gender versus model. We observe that the effect of gender does not have a statistically significant influence on the WER.

Source	SS	DF1	DF2	MS	F	p-unc	np2	eps
Gender	0.000674	1	38	0.000674	0.026105	0.8725	0.000686	-
Model	1.022612	3	114	0.340871	143.958631	**1.295907e-38**	0.791161	0.708692
Interaction	0.002457	3	114	0.000819	0.345837	7.922205e-01	0.009019	-

Bold p-vlues indicate statistically significant results.

Figure 1 shows the performance of the different speech recognition systems across all groups based on Language and Gender. We observe from the box plots that the Word Error Rate of Whisper is the lowest of all 4 groups, followed by Microsoft and Google, whilst Vosk performs the worst for all 4 categories. The detailed Shapiro-Wilk test for normality, pairwise t-tests, and box plots of all pairwise comparisons between the respective models are provided in Supplementary Material S4.

**FIGURE 2:**
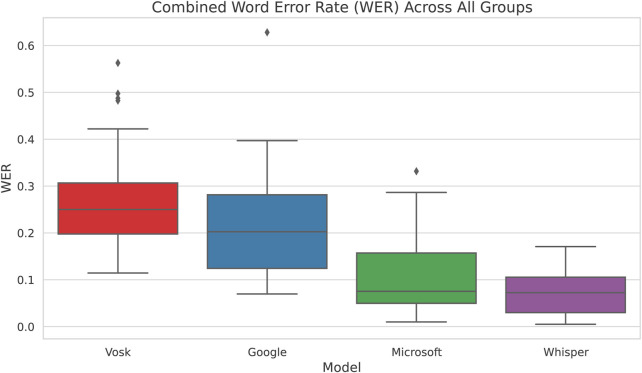
Word Error Rate vs Speech Recognition System for all Participants. We see that Whisper has the lowest WER and variability compared to the other models.

Combining participants of all groups and analysing the Word Error Rate, we see in Figure 2 that Whisper has the least WER across followed by Microsoft and then Google. The pairwise t-test in Supplementary Material S4 confirms these results as well by showing that Whisper is significantly better than the other 3 models when data from all groups are used to evaluate the models. Based on these results, we conclude that, based on the experiment conducted, Whisper is the most accurate model in terms of ensuring language and gender parity.

## 4 Methods - language models and large language models

After making strides towards making our agent equitable, we move on to proposing a novel multi-LLM architecture in order to try and obtain faster, cheaper and more customisable solutions that could potentially rival the state-of-the-art - the custom GPTs by OpenAI OpenAI (2023). We begin by introducing the popular models used in the study, the methods incorporated to tune and obtain the necessary results and the questionnaire used to evaluate our model against the state-of-the-art.

### 4.1 Relevant models

Here we discuss the popular models that are used as a part of our work. We also introduce the state-of-the-art - the GPTs.

#### 4.1.1 Bidirectional encoder representations from transformers (BERT)

BERT is a language model developed by Google, with significant applications in the field of natural language processing. Unlike previous models that read text unidirectionally, BERT processes text in both directions simultaneously. This bidirectional approach allows the model to capture a more nuanced understanding of context, making it highly effective at understanding the meaning of each word in its textual environment. BERT is pre-trained on a large quantity of text from the internet, including the entire Wikipedia, which enables it to learn a wide range of language patterns and structures. Furthermore, it can be fine-tuned with additional data for specific tasks without substantial modifications to the underlying model. This versatility has led to its widespread application across a variety of NLP tasks such as text classification, question answering, sentiment analysis, and named entity recognition, revolutionising how machines understand human language Devlin et al. (2018). Furthermore, works such as Sanh et al. (2020) have proposed methods of shrinking the model size by 40% whilst retaining 97% of the accuracy.

#### 4.1.2 GPT 3 and GPT 3.5 turbo

The GPT-3 and GPT-3.5 Turbo series, developed by OpenAI, includes a range of language models designed for generating text that closely mimics human language for chat applications. The foundational model in this series, known as davinci, is equipped with 175 billion parameters, showcasing its proficiency in text generation. OpenAI has enhanced the capabilities of davinci through two primary development paths. The first path involves supervised fine-tuning, leading to the creation of InstructGPT, also referred to as text-davinci-001. The second path focuses on training for coding tasks, resulting in the Codex model. The evolution continued with the release of code-davinci-002 in 2022, specifically designed for code generation, which laid the groundwork for the GPT-3.5 series.

Further advancements led to the development of text-davinci-002 through additional supervised fine-tuning, alongside the introduction of the RLHF (Reinforcement Learning from Human Feedback) ? training strategy with text-davinci-003 Brown et al. (2020). This new strategy significantly enhanced the model’s ability to interpret instructions and produce relevant text. Building on the success of text-davinci-003, OpenAI optimised a model specifically for chat applications, known as gpt-3.5-turbo. This model stands out for its high capability at a more affordable cost compared to its predecessor, text-davinci-003.

Although other open-source models such as LLaMA 13b, Mixtral 8 × 7b and Mistral 7b exist, for this study we use GPT 3.5 Turbo since it is easy to use, is extremely reliable, has good documentation, allows for fine-tuning directly on their website with optimised hyperparameter setting and does not need any specific GPU for training and inference. The other evaluated models do not provide all of these merits under a single umbrella at the time of writing this paper.

#### 4.1.3 GPTs - the state-of-the-art

In November 2023, OpenAI presented a novel approach to LLM customisation by empowering users to tailor specific instances of ChatGPT for particular tasks. This feature enables the customisation of digital assistants for a wide range of applications, from learning and productivity to entertainment and scientific research, without requiring any coding skills. With an emphasis on the importance of community involvement in the development of GPTs, it highlights the potential for users to contribute to the diversity and capability of these tools. Furthermore, with the launch of the GPT Store, creators will have the opportunity to share their GPTs with a broader audience, potentially earning revenue based on usage. The GPTs come with built-in functionality such as image generation using DALLE3, web browsing using Bing, code writing and execution, knowledge retrieval and even advanced function calling and customised actions. We argue that the versatility and access to powerful resources make GPTs a powerful benchmark to evaluate even highly niche chatbots. Before the release of GPTs, chatbots did not have a standard one-size-fits-all state-of-the-art since they were extremely task-specific. This innovation, however, enables a quick and powerful agent powered by an LLM to serve as a reference to compare and evaluate diverse chatbot technologies OpenAI (2023).

### 4.2 Popular methods used for implementing LLMs

After discussing the popular models used in this study, we go over the different methods and approaches implemented to improve the capabilities and tune our models for our required goals by drawing inspiration from related discoveries and innovations in the field of Large Language Models and information retrieval.

#### 4.2.1 Chain-of-thought prompting and reasoning

Chain-of-Thought (CoT) prompting guides large language models through a series of intermediate steps or thoughts towards a solution, mirroring human problem-solving processes. This technique enhances the models’ capacity for complex reasoning across tasks, from arithmetic to commonsense reasoning, by making their decision-making pathways more transparent and interpretable. CoT prompting has proven especially effective in improving performance without extensive task-specific fine-tuning, highlighting its utility in leveraging pre-trained models for a broader range of applications while maintaining or enhancing their accuracy and interoperability Wei et al. (2023).

CoT is useful when directly reaching a particular answer is difficult. Akin to teaching a child how to solve a given problem, CoT provides a reliable framework to break the problem into parts and solve it sequentially and in a methodical fashion. Chain-Of-Thought can also make the underlying motivations and reasons behind LLMs choices more transparent and easier to tune based on the specific goals.

#### 4.2.2 Fine-tuning LLMs

Fine-tuning in the context of large language models is the process whereby a pre-trained model is adjusted to perform a specific task better. This process involves taking a model that has been trained on a large, general dataset and further training it on a smaller, task-specific dataset. The rationale behind fine-tuning is that the pre-trained model has already learned a vast amount of general knowledge about the language, and fine-tuning allows it to adapt this knowledge to the requirements of a particular domain. This is achieved by adjusting the model’s weights based on the task-specific data, often involving a lower learning rate to make small, incremental changes that refine the model’s abilities without overwriting its pre-existing knowledge. Fine-tuning is critical in a wide variety of tasks such as sentiment analysis, question-answering, and text classification, enabling models to achieve high performance with relatively less task-specific data.

Parameter-Efficient Fine-Tuning (PEFT) methods, such as Low-Rank Adaptation (LoRA), and its quantised variant, q-LoRA, represent advanced strategies to reduce the computational and memory burden associated with fine-tuning LLMs. These techniques focus on modifying a small subset of the model’s parameters or introducing additional parameters that can learn task-specific features without altering the entire model. LoRA, for example, introduces low-rank matrices that interact with the pre-trained weights to adapt the model’s output without directly modifying the original weights. This allows for efficient adaptation to new tasks while keeping the majority of the model fixed, significantly reducing the required memory and computational resources. q-LoRA extends this by applying quantisation to the adaptation process, further decreasing the computational load and storage requirements. These methods exemplify the shift towards making fine-tuning more accessible for a wider range of applications, especially in resource-constrained environments Xu et al. (2023).

Fine-tuning enables the application of LLMs to a broad spectrum of tasks while leveraging their pre-trained general knowledge, thus bypassing the need for training large models from scratch for every new task. This adaptability significantly lowers the barrier to entry for deploying state-of-the-art models in specialised domains. Moreover, fine-tuning can lead to models that are not just more efficient but also more accurate, as they can be tailored to the peculiarities of a specific task or dataset. Fine-tuning can also be employed to teach models specific formats of output or to respond in a particular manner making the results more deterministic than their pre-trained predecessors.

#### 4.2.3 Retrieval augmented generation (RAG)

Retrieval-Augmented Generation (RAG) is a technique used to integrate the capabilities of large language models with external knowledge bases to allow the model to access task-specific information and generate informed and accurate responses. Without RAG, models are restricted by the knowledge they learnt during pre-training. Any information provided after the cut-off date is unavailable to the model and questions regarding the same are often answered incorrectly with hallucinated answers. RAG involves retrieving relevant documents from a database based on the input query and using this information to guide the generation process, allowing the model to produce contextually relevant responses Gao et al. (2024).

The primary benefits of this approach include the model’s improved capacity to incorporate up-to-date information, a reduction in generating inaccurate or fabricated information (hallucinations), and the ability to access domain-specific knowledge beyond its original training data. Hallucinations are reduced by grounding the LLM to respond only based on the information provided to it and prompting it to respond with ‘I don’t know’ when the necessary information is not available Gao et al. (2024).

#### 4.2.4 Retrieval augmented fine tuning (RAFT)

Retrieval Augmented Fine Tuning (RAFT) is a methodology that enables the integration of external knowledge into language models during their fine-tuning phase and it is shown to be a powerful approach to derive the best of both worlds for enhancing the capability of LLMs to understand and respond to queries within specific domains. RAFT works by initially retrieving a set of documents, D from a knowledge base relevant to a given query. These documents are selected based on their potential relevance to the query’s context. Then, using Chain of Thought reasoning, the model evaluates these documents to identify a subset, D* that is most relevant to the asked query. This fine-tuning process involves teaching the model to differentiate and prioritise information from D* that significantly contributes to generating accurate and contextually appropriate responses. This approach enables the model to leverage external knowledge effectively, enhancing its capability to address complex, domain-specific inquiries.

The concept of RAFT is explained with a nice analogy in Zhang et al. (2024). While RAG is akin to a student in an open book exam who has not prepared for it and fine-tuning is akin to a student in a closed book exam who has learnt the subject matter well, RAFT provides the alternative, of a case where a student has prepared for an open book exam and can effectively utilise the resources at their disposal during the test.

### 4.3 Evaluation questionnaire

For the evaluation of our conversational agent, we use the Artificial Social Agent Questionnaire (ASAQ) Fitrianie et al. (2025). The questionnaire was developed based on the need to create a validated, standardised measurement instrument dedicated to assessing human interaction with Artificial Social Agents (ASA). The ASAQ is the result of extensive collaboration over multiple years involving over one hundred ASA researchers globally and ensures a robust framework for evaluating interactions between humans and ASAs. The long version of the ASAQ provides an in-depth analysis of human-ASA interactions, catering to comprehensive evaluation needs. Conversely, the short version offers a swift means to analyse and summarise these interactions, facilitating quick insights into the user experience. Additionally, the instrument is complemented by an ASA chart, which serves as a visual tool for reporting results from the questionnaire and provides an overview of the agent’s profile. Due to its breadth and comprehensiveness, the ASAQ measures 19 parameters–some of which are not relevant to our study. We go over all 19 criteria and the 13 relevant ones used in our study, featuring the short version of the ASAQ in Supplementary Material S10.

## 5 Design of the multi-LLM conversational agent

Now that we have set the stage for the proposed solution, we will cover the main requirements, design strategies and specific details of how we built a multi-LLM agent. A supermarket chatbot must be capable of retrieving relevant information from the supermarket database, answering user queries in a friendly and natural manner whilst ensuring it can handle a variety of user queries from simple requests asking details about a specific product (e.g., “Where can I find Oreos and how much are they?”) to complex high-level queries (eg., “I am not sure of what to make for dinner, can you recommend some ideas and the necessary ingredients?”). This requires the conversational agent to not only be capable of the basic functions such as natural language understanding, dialogue management and natural language generation, but also advanced reasoning and information retrieval.

The current state-of-the-art for handling all these different responsibilities is the concept of GPTs OpenAI (2023). However, GPT4 Turbo, the underlying model in GPTs, is not without its own set of limitations. Firstly, for applications such as chatbots, latency is an extremely important factor. Despite recent advances in speed, GPT4 is significantly slower in response generation compared to smaller and lighter models due to the overall size of the model, which results in slower inference speed. Furthermore, research has also indicated that for extremely large context windows GPT4 tends to struggle with information retrieval. This form of evaluation–titled needle in a haystack is used to measure how well a model can retrieve information based on the position of the requested information in the overall context Kuratov et al. (2024). Due to the limitations in model architecture, the model struggles with correctly retrieving the information in the middle portions of the context when the context size reaches close to the limit. Furthermore, there are also claims that the reasoning window of powerful LLMs is much lower than their context window, making the task of advanced reasoning over large amounts of information significantly challenging. Lastly, the model is significantly more expensive at the time of writing this paper, at $10 per million tokens input and $30 per million tokens output, than the cheaper GPT-3.5 Turbo priced at $0.5 per million tokens input and $1.5 per million tokens output. Even after fine-tuning, the GPT 3.5 Turbo model costs $3 per million tokens input and $6 per million tokens output–a significant reduction over the GPT4 non-finetuned counterpart.

**FIGURE 3:**
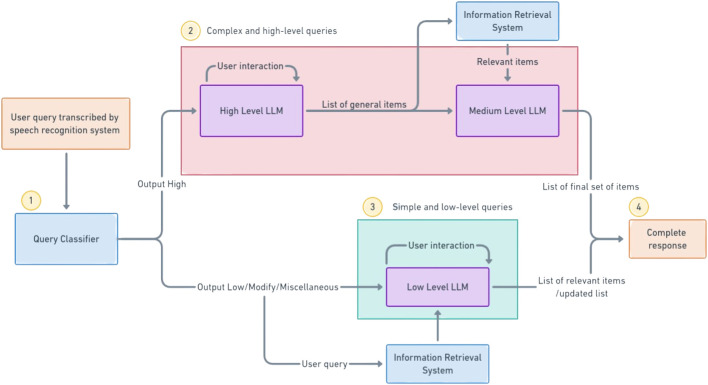
Proposed architecture for handling different queries. Once the query has been transcribed by the speech recognition system, it is classified by the mdistilBERT system (1). If the query is classified as a high-level query, the high-level LLM asks further questions and prepares a rough list of items. These items are sent to the information retrieval system and the relevant items are sent to the medium-level LLM that prepares the correct list of items (2). Otherwise, the query is directly converted to an embedding and searched by the IR system to provide the necessary list of items to the user (3). The relevant response (4) is then shown to the user for further modifications or approval.

To address the problems of latency, information retrieval, reasoning window and price, we propose a novel multi-LLM conversational agent where many smaller LLMs, specialised for certain tasks and query types work together to give better results. For our implementation, we use multiple GPT-3.5-Turbo models in a hierarchical fashion where each model serves a specific purpose. Furthermore, the input query is classified using distilBERT into high-level or low-level queries, allowing for a different strategy to be employed for different query complexity, further optimising computational resources and API costs. The architecture we employ is shown in Figure 3.

### 5.1 Query classifier

The first step in our conversational agent is to take the input text obtained from the speech recognition system and classify it based on whether the query is high-level, low-level, modification or miscellaneous. The types of queries are explained below:

1. High-level queries are those that need to be broken down and analysed with the help of the user to ascertain their preferences, the particular occasion and other restrictions which can enable us to make more informed decisions.
2. A low-level query is a specific request for a particular product or class of products, such as finding the location, price or alternatives to an option,
3. A modification query is one where the customer wishes to make amendments to a previously displayed list.
4. A miscellaneous statement comprises everything else, such as conversational statements like “Yes, please” and “thank you.”

To classify these requests, however, we need a powerful natural language classifier that can be fine-tuned for the given task. The classifier we chose to proceed with is a condensed form of the Bidirectional Encoder Representations from Transformers (BERT). We used distilBERT Sanh et al. (2020)–a general-purpose model that reduces the size of conventional BERT by 40% while retaining 97% of the task performance and can be run locally on systems without dedicated GPUs. The model is freely available on HuggingFace and is easy to train and deploy. The query classifier is trained on over 150 examples–augmented by GPT4 by providing a few representational examples to the model. Furthermore, to also support Dutch, we use the multilingual version of distilBERT.

For our fine-tuning purposes, we were able to use anonymous logs of chatbot interactions in previous experiments, along with GPT4 augmented data. In total, we had 106 English statements, manually labelled from the previous chatlogs and 250 English queries were augmented by GPT4. The data augmentation was done on the ChatGPT interface to allow for better control of the diversity and nature of resultant statements. These 356 queries were translated to Dutch whilst respecting the conversational nature of the queries by GPT4. After shuffling the data, we split the final 712 queries into 500 training, 106 validation, 53 English tests and 53 Dutch test sets. Before training the queries were converted to lowercase and punctuation marks were removed since we are using a cased distilBERT model.

The hyperparameters used are as follows -

1. Learning rate: 5e-5,
2. Number of epochs: 8,
3. Optimiser: AdamW,
4. Warmup steps: 10% of total steps

The final validation loss was 0.58324 and the final validation accuracy was 0.8302 at the 8th epoch and was unchanged from the 7th epoch results. The table summarises the accuracy, recall, precision and F1 scores of the classifier after fine-tuning. The fine-tuned mDistilBERT classifier demonstrates a robust performance in classifying queries into four distinct classes: high, low, modify, and miscellaneous, with both English and Dutch test sets achieving comparable accuracy scores of 0.8679. This similarity in accuracy suggests that the model generalises well across languages, a testament to the multilingual capabilities of the underlying mDistilBERT architecture. Precision scores, slightly higher for the English set at 0.8839 compared to 0.8710 for Dutch, indicate a marginally better reliability in the model’s positive predictions for English. The recall scores, identical for both languages, affirm the model’s effectiveness in identifying relevant instances across the dataset. However, the F1 Score, which balances precision and recall, is slightly higher for the English test set (0.8651 vs. 0.8635 for Dutch), suggesting a modestly more balanced performance in English. Overall, these metrics reflect the classifier’s proficient handling of varied linguistic queries, illustrating its practical utility in multilingual applications. The results are summarised in Table 4.

**TABLE 4:** Performance metrics for english and dutch test sets for query classification by mDistilBERT.

Metric	English				Dutch			
	Accuracy	Precision	Recall	F1 Score	Accuracy	Precision	Recall	F1 Score
Value	0.8679	0.8839	0.8679	0.8651	0.8679	0.8710	0.8679	0.8635

The slight differences between English and Dutch performances could offer insights into areas for further model optimisation, particularly in enhancing its cross-linguistic adaptability and understanding. While the performance may not seem remarkable, it is important to note that the mistakes made in classification are sometimes permissible. For example, in the English test set the classifier mislabeled “Sure, add that to my cart.” as “modify” instead of the ground truth label assigned of “miscellaneous,” which is a completely valid classification for the given query. Likewise, the dutch query, “Ik moet mijn gebruikelijke ontbijtgranen vervangen door een optie met veel vezels, welke?” (translation - I need to replace my usual breakfast cereal with a high-fiber option, which one?) was misclassified as a high-level query when the ground truth label assigned was low–which is once again a permissible misclassification since there are multiple options for a high-fibre breakfast (high level) but it can also be a low-level query (retrieve the high-fibre cereal options). Thus, we argue that the performance of the classifier is better in true application than the results indicate.

### 5.2 Personalisation

To understand what information to capture from the user, we draw inspiration from Sharma and Levy (1995), which presents the different categories into which retail salespeople cluster customers. We argue that since the conversational agent effectively replaces the salesperson in the supermarket, the same categorization can be used to create an effective user profile. The 6 quantitative parameters we capture on a Likert scale from 1-5 are:

1. Price Consciousness: the measure of how much the customer cares about cheaper products, substitutes, sales and discounts over premium products.
2. Brand Loyalty and Value: the measure of the customers’ tendency to stick to certain premium product brands rather than allowing other parameters like price or size to affect their decision.
3. Help Appreciation: the degree to which the customer values recommendations and help from external sources in the supermarket.
4. Degree of knowledge: the degree to which the customer believes they are knowledgeable about the various products available in the store and thereby how specific or broad their requests may be.
5. New product exploration: the degree of willingness to try out new products and offerings instead of continuing to rely on more standard and predictable patterns while shopping.

The 2 qualitative questions we ask users are:

1. Dietary preferences to understand any allergies, specific habits or principles followed.
2. Product interest which involves any specific information about any products or brands they prefer purchasing or exploring.

We believe that this information is key for the chatbot to provide personalised and useful recommendations to the user whilst also tailoring the conversation in a manner beneficial to the customer. This information can then be retrieved when the customer scans their membership card (for example) and fed as context to an LLM to enable it to make more informed decisions and provide personalised recommendations.

### 5.3 Product database

Due to the absence of a simple and relevant database comprising of different categories in a supermarket, the products, their prices, potential discounts and locations, a dataset was augmented using GPT4 with the ChatGPT interface. ChatGPT was used instead of completely automating the process via API call for lower costs and greater control over the number of products per category and toggling the price/discount if necessary. Overall, we had 100 different categories comprising standard grocery, personal care, home maintenance, tools, electronics, books and furniture to name a few. Each category had anywhere from 12 to 30 products created along with brand names, prices, discounts and shelf numbers. Thus, overall 1612 products were augmented by GPT4, which will be used as the dataset to demonstrate the functioning of our system. The same database will also be provided to the GPT made via OpenAI so that both approaches have access to the same ground truth. This also helps serve as a reference to evaluate the number of hallucinations by the models since not grounding them could result in these systems making up completely new items that do not exist in the current inventory.

### 5.4 Information retrieval

Next, we will discuss the process of information retrieval carried out in our approach. Firstly, we transform the original data into an inverted index where each product, its location, price, and potential discounts occupy one row. After this, we use an embeddings model (text-embedding-3-small by OpenAI) to convert all 1612 products into n-dimensional vectors that are then stored as a NumPy file. We then convert either the low-level query or all elements of the medium-level LLM response into an embedding via the same model and find the closest neighbours using cosine similarity. For low-level queries, we retrieve 20 closest products to allow for sufficient recall and for each element in the medium-level LLM response, we retrieve 3 closest products. Thus, if the medium tier LLM returns 7 items, we will retrieve 21 products based on the closeness of these items to their neighbours.

### 5.5 High-level LLM

If the query is classified as high-level, a high-level LLM is called to interact with the user in order to get more information and break down the query into a list of items the user may need. At this step, user preferences and choices are taken into account, along with ascertaining what items the user would need versus those which they already possess or can be substituted. This is best explained with an example. Say you want to bake a cake. There are several ingredients you need, such as milk, eggs, flour, baking powder, baking soda, vanilla essence, sugar, etc. However, you may possess a lot of these items already at home. Additionally, there are other ways to make a cake, such as using a cake mix, buying a premade cake or deciding exactly what flavour and nutrition profile you wish to base it on.

The high-level LLM is tasked with ascertaining what kind of cake you want, if you have any preferences/allergies or other customisations needed along with understanding the exact list of ingredients you would need. The high-level LLM is a GPT3.5 Turbo fine-tuned on 36 multi-turn conversations using previous chatlogs of preliminary user interaction and context-relevant conversations augmented using GPT4 via ChatGPT. A validation dataset is also created, comprising 12 similar conversations. Overall, the fine-tuned model after 3 epochs has a training loss of 0.6228 and an accuracy of 0.80365, while the validation loss is 1.0575 and validation accuracy is 0.56343. This indicates that the model has overfitted on the training data, however, given the complex multi-turn nature of the task, even considerable deviations from the ground truth presented in the validation data are possible due to the likelihood of having multiple correct answers to a query based on user responses. The high training accuracy indicates that the elements we care about, such as the format and the approach, have been well learnt.

### 5.6 Medium-level LLM

Once the user is satisfied with this selection of items, the list of user-selected products, the chatlog of the user and the chatbot, the user profile and the retrieved items are sent to a medium-level LLM that is tasked with creating a tailored list of items from the context with the exact name, brand, price, location and reasoning behind the selection of the items. The medium-level LLM never interacts directly with the user. Based on the response of the medium-level LLM, the user can fine-tune their list of items by making any final changes to the products using the low-level LLM.

The fine-tuning of this model draws inspiration from Retrieval Augmented Fine Tuning (RAFT) Zhang et al. (2024). RAFT provides a simple approach to derive the best of both Retrieval Augmented Generation and fine-tuning. The essence of RAFT lies in providing D retrieved documents or relevant pieces of information (in our case, product details) and fine-tuning the model to use Chain-Of-Thought reasoning to select D* relevant items. For instance, if 5 different types of flour are retrieved and used as context by the LLM, we specifically use Chain-Of-Thought reasoning to select the whole wheat flour if the user profile indicates that the customer is health-conscious. This way, we are not only able to fine-tune our model to present the results in the right format but also can teach it how to select the most relevant items from a larger pool of options. We use GPT4 for automating the process of constructing the chain of thought reasoning and selecting the relevant items, which are then manually verified to ensure there are no significant mistakes or issues. Using GPT4 for this process also enables us to leverage its ability to write neat responses with sensible changes in text font, correct usage of bold and italics and even-numbered/unnumbered lists. Furthermore, since GPT4 provides the entire chain of thought reasoning as the response, the whole process can be automated, including the creation of the jsonl files and finetuning of the model after their validation.

Our model is fine-tuned on 25 training and 5 validation single-step conversations using the GPT 3.5 Turbo model. The provided contextual chatlogs are taken from the training set of the high-level LLM whilst maintaining the same user profile. We achieve a training loss of 0.64398, training accuracy of 0.81544, validation loss of 0.80797, and validation accuracy of 0.692. The differences between validation and training are smaller in this case since the conversations are not multi-turn. However, even in these conversations, there are differences in the retrieved context from the database and some degree of changes in the responses are to be expected.

### 5.7 Low-level LLM

Should the user ask for a low-level, modified or miscellaneous query or remark, we call a low-level LLM capable of retrieving the information from the database and giving the output to the user, whilst also editing the bill based on the specific request. The process continues until the user is happy with their list and there are no further edits or changes necessary. The low-level LLM receives 20 products from the information retrieval system after converting the original query to an embedding and finding the closest neighbours via cosine similarity.

Similar to the strategy employed in the medium-level LLM, we use RAFT to provide chain-of-thought reasoning during fine-tuning to ensure the correct and most relevant items are picked from the larger pool. The responses are once again created by GPT4 and manually verified to ensure reliability and consistency. The other benefits, such as format and style are thus applicable even for this chatbot.

Our model is fine-tuned on 40 training and 10 validation single-step conversations using the GPT 3.5 Turbo 0125 model. The provided contextual chatlogs are taken from the training set of the high-level LLM whilst maintaining the same user profile. We achieve a training loss of 0.17557, training accuracy of 0.94014, validation loss of 0.88602, and validation accuracy of 0.67705. Once again, we observe considerable differences between the evaluation of the training and validation datasets, which can once again be justified by the complexity of the conversations and the existence of multiple correct answers. These results are better than the high-level LLM as there is still some amount of grounding due to the usage of relevant items.

**FIGURE 4:**
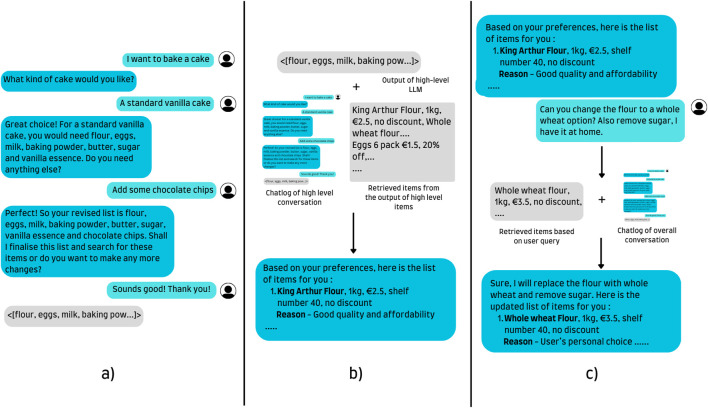
A visual depiction of the responses of the 3 different LLMs. A high-level query takes a request and, based on the user’s input and user profile, creates a basic list of items. The medium-level LLM takes these items, the chatlog and retrieved items to craft a tailored response for the user. Lastly, all specific queries, modifications and other requests are passed to the low-level query capable of retrieving items and making changes to the original list. **(a)** High level LLM. **(b)** Medium level LLM. **(c)** Low level LLM.

All three models have been trained on English conversational data. While training a multilingual model on only English data is not ideal and free of bias, the same choice is supported by the intrinsic ability of LLMs to converse in multiple languages and limitations in the author’s proficiency in Dutch. A simple overview with an example of the different models working side by side is provided in Figure 4. The relevant prompts used for the high-level, medium-level, low-level and supermarket data augmentation are available in Supplementary Material S11.1, S11.2, S11.3, S11.5, respectively. Furthermore, the demonstration of our approach featuring the onboarding and different LLMs used at different stages of the conversation has been covered in Supplementary Material S14.

### 5.8 Text-to-speech system

The final component of a conversational agent is the Text-To-Speech (TTS) system, which converts output text into a spoken voice. This study does not evaluate various TTS systems, as user preferences regarding accent, gender, and clarity vary widely. Therefore, the ideal system should offer users options based on their preferences. In this work, we utilize the OpenAI Text-to-Speech system (Echo), as demonstrated in Supplementary Material S14. OpenAI’s TTS is available in multiple voices–Alloy, Echo, Fable, Onyx, Nova, and Shimmer–and produces extremely natural-sounding speech. It can also recite lists in a friendly, human-like manner. However, it is a premium service, currently priced at $15 per 1 million characters. For those seeking a cost-free option, Google’s text-to-speech system may be more viable. While free, its output tends to sound more robotic, which may not be ideal for real-world applications. It is also important to note that currently, none of the major industry players, such as OpenAI, Google, and Microsoft, offer a Dutch TTS system that is both natural-sounding and available in multiple genders via an API. However, some recent AI startups, such as ElevenLabs?, now provide multilingual text-to-speech capabilities, including Dutch, making them a viable option for supporting multilingual speech.

## 6 Evaluation of our architecture against the state-of-the-art

While conversational agents are often built for specific tasks and the existence of a state-of-the-art is difficult to justify, the emergence of GPTs and Assistants API by OpenAI provides a great benchmark to compare our architecture against. In November 2023, OpenAI released GPTs a novel concept allowing people to create their own custom AI models built on top of the GPT4 Turbo model. They are capable of image creation, code generation, knowledge retrieval, function calls, etc., and can be developed with absolutely no code. It is a revolutionary new step in democratising AI development and usage by allowing everyone to build, share and even earn from the GPTs they create. Assistants API extends the same concept to a business perspective by allowing these GPTs to be hosted on the websites of the host. These models use the latest developments in information retrieval and the state-of-the-art LLMs to perform the necessary tasks with great accuracy. The details of the specific GPT created for the scope of this experiment can be found in Supplementary Material S12, including the instructions provided and the enabled functionalities.

However, currently building and deploying chatbots using the Assistants API is extremely expensive due to the high token consumption by these models for knowledge retrieval tasks. Furthermore, the current setting allows for using only a single model per assistant preventing task optimisation based on specific goals. GPTs, although a part of the Plus membership of ChatGPT cannot be currently used outside the ChatGPT interface. Also currently being in the beta phase, it is prone to malfunctioning as new features and evaluations are still ongoing. However, it still serves as a strong contender and we use it to benchmark the performance of our custom multi-LLM chatbot against it. To do this, we perform a within-subjects experimental study where participants are split into two groups based on the order in which they try both chatbots. We ask both groups to fill out the Artificial Social Agent Questionnaire (short version) with all relevant questions and also ask certain qualitative questions to understand their overall experience. We then compare the ASAQ scores of both groups to ascertain which model is ranked better by users on all relevant metrics measured by the questionnaire. The within-subjects design factor ensures all participants try both models and thus can also provide qualitative feedback on their overall experience.

### 6.1 Participant demographics

Overall, 16 participants were recruited for the study (9 male and 7 female) between the ages of 23–30 (Mean - 24.3125 and SD - 1.8874). In terms of frequency of usage and familiarity with LLM chatbots like ChatGPT, Gemini and Claude, 6 participants responded that they interact with such tools over 5 times a week, 3 responded between 4–5 times a week, 2 responded 3–4 times a week, 4 responded 1–2 times a week and 1 participant responded less than once a week.

### 6.2 Experiment design

All participants were first shown the informed consent form to reassure them that no personally identifiable information would be collected (Supplementary Material S13). The only data stored are their responses to the questionnaire, answers to the qualitative questions and chatlogs for further analysis of factors such as hallucinations. We began by collecting demographic details and asking for a brief insight into their shopping intentions, such as what they look for and prioritise when they are shopping in the supermarket. Participants were then asked to interact with either the GPT or the custom multi-LLM chatbot we created. The order in which participants tried both chatbots was routinely cycled to ensure half the participants started by interacting with the GPT and the other half with our solution. Participants were not informed of the nature of the agents and were asked to interact with them in a manner they felt best expressed their supermarket intents and goals. After interacting with the first chatbot, participants were asked to fill in the 13 relevant questions from the ASAQ followed by the following qualitative questions -

1. Tell us in detail, what do you find most helpful and unhelpful from this result.
2. If at all, how much does this system make you feel more or less confident about your shopping needs and decisions in a supermarket?
3. Is there anything that you would like to comment about this task?

After this, they were asked to repeat the same procedure but with the other chatbot. The overall experiment took roughly 40 min to complete.

Since order is the between-subjects factor and the chatbot is the within-subjects factor, we perform the Mann-Whitney U-test and the Wilcoxon signed-rank test, respectively. We use these non-parametric tests since the Shapiro-Wilks test of all the criteria was not normally distributed. This is to be expected given that we were using ordinal data as opposed to continuous values.

### 6.3 Questionnaire results

**FIGURE 5:**
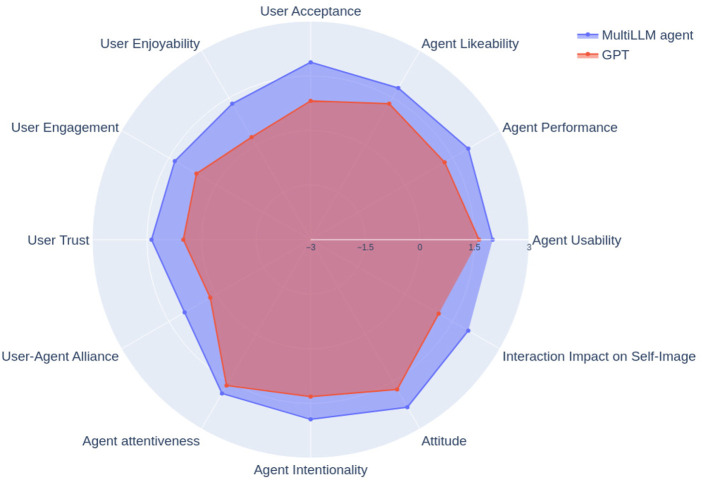
Comparison of the GPT with our custom multi-LLM solution on the provided ASA chart. The scores range from −3 to +3 on the Likert scale on which the ASAQ is built. Our multi-LLM approach performs better than the GPT on all 13 parameters.

As seen in Figure 5, we observe that our solution performs better than the GPT on all 13 tested parameters of the ASAQ. We continue by performing statistical tests on all 13 parameters to find out which parameters are significantly better in our model compared to the state-of-the-art. Table 5 lists all 13 parameters. Overall, we observe that in terms of agent performance, user acceptance of the agent, user-agent alliance, agent attitude and interaction impact on self-image, the p-value is less than 0.05. The Mann-Whitney U-test shows that order is not statistically significant for all criteria except the agent’s attitude. Thus, we cannot rule out the agent’s attitude as being statistically better since order could have influenced the results.

**TABLE 5:** Summary of statistical analysis of the artificial social agent questionnaire. Variable μ is the mean value and variable σ is the standard deviation.

Sl. No	Criterion		Group scores				Statistical tests	
			GG	GC	CG	CC	WSR	MWU
1	Agent’s Usability	μ	2	1.875	1.25	2.125	p = 0.19	p = 0.50
		σ	0.7559	0.6408	1.0351	0.3535	W = 12.0	U = 144.5
2	Agent’s Performance	μ	1.5	1.75	1	2.25	p = **0.048**	p = 1.00
		σ	1.1952	0.8864	1.0690	0.4629	W = 15.0	U = 128.0
3	Agent’s Likeability	μ	1.5	1.75	1.125	1.875	p = 0.299	p = 0.814
		σ	1.4142	0.7071	1.4577	1.1260	W = 36.5	U = 134.5
4	User Acceptance of the Agent	μ	1.125	1.75	0.5	2	p = **0.022**	p = 1.00
		σ	1.1260	1.0350	1.8516	1.3093	W = 13.5	U = 127.5
5	Agent’s Enjoyability	μ	0.25	1.25	0.25	1.375	p = 0.091	p = 1.00
		σ	2.0528	1.8322	1.7525	1.5059	W = 26.0	U = 127.5
6	User’s Engagement	μ	1	1.75	0.25	0.875	p = 0.095	p = 0.082
		σ	1.1952	0.7071	0.8864	1.8851	W = 26.5	U = 173.0
7	User’s Trust	μ	1	1.25	0	1.5	p = 0.104	p = 0.63
		σ	1.3093	1.0351	1.5118	1.7728	W = 22.5	U = 173.0
8	User-Agent Alliance	μ	0.75	1.125	−0.375	0.875	p = **0.027**	p = 0.065
		σ	0.7071	0.8345	0.9161	1.7268	W = 0.0	U = 0.065
9	Agent’s Attentiveness	μ	1.625	1.75	1.625	2	p = 0.484	p = 0.633
		σ	1.1877	0.7071	1.0606	0.5345	W = 30.5	U = 115.5
10	Agent’s coherence	μ	2	1.75	0.625	2.125	p = 0.108	p = 0.292
		σ	0.7559	1.1650	1.4079	0.8345	W = 19.0	U = 155.0
11	Agent’s intentionality	μ	2.125	2.125	1.375	2.5	p = 0.087	p = 0.732
		σ	0.6409	0.9910	1.0606	0.7559	W = 6.0	U = 137.0
12	Agent’s attitude	μ	1.625	2.25	0.5	1.75	p = **0.022**	p = **0.048**
		σ	0.9161	0.7071	1.6036	0.7071	W = 8.0	U = 178.0
13	Interaction Impact on Self-Image	μ	0.875	1.5	0.25	1.5	p = **0.017**	p = 0.694
		σ	0.8345	1.0690	1.5811	0.9258	W = 10.0	U = 138.5

The 4 groups mentioned are an order model pair and stand for: GG - GPT first GPT scores, GC - GPT first Custom chatbot scores, CG - Custom chatbot first GPT scores and CC - Custom chatbot first custom chatbot scores. All 13 criteria fail the Shapiro Wilks test for normality and thus the Wilcoxon Signed Rank test (shown in the table as WSR) is done to evaluate the performance between models with p-value and Wilcoxon statistic represented as W and Mann-Whitney U-test (shown in the table as MWU) is performed to test the effect of order with p-value and Mann Whitney statistic represented as U are presented below. Bold p-vlues indicate statistically significant results.

### 6.4 Qualitative results

As mentioned in the experiment design, participants were also asked 3 qualitative questions to try and understand their overall experience better.

#### 6.4.1 Benefits of GPT

Participants overall agreed that the GPT model was simple to use and interact with. Furthermore, all participants who were looking for detailed recipes and instructions on making certain meals and dishes were extremely pleased with the detailed responses of GPT. Participant #5 commented on its usefulness as a brainstorming tool to help make decisions about what to purchase and what to try out. Participant #7 found the responses of the GPT to be more cohesive and in line with their expectations when inquiring about meal preparation strategies for the entire week. Furthermore, participant #15 found that the responses to complex questions were quite well handled whilst ensuring the conversational tone and language were simple to understand. Whilst none of the participants were overly enthusiastic about the responses and strength of this system, they were content with the answers and recommendations provided by it.

The different merits of the GPT model can be attributed to the agent’s powerful underlying model (GPT4 Turbo), its reliability in keeping track of previous conversations with relative ease and its flexibility to handle all kinds of queries even those that diverge from the traditional product recommendation and information objective (e.g., recipes or detailed plans to achieve a goal). This makes the agent more robust to greater customer variations in requests whilst also having a track of all the conversations with the user in mind.

#### 6.4.2 Concerns about GPT

Participants #2 and #3 were concerned about hallucinations and mentioned that this affected the degree of trust they could place in the system. P#2 found some items which did not exist in the database in the misleading responses (hallucination), whilst P#3 was not able to get information about a screwdriver despite the item being present in the database (omission). Participant #6 had issues substituting organic spinach with regular spinach despite several attempts. Participants #4 and #8 found the number of options provided by the GPT was limited which made them feel more restricted in terms of choices. Participant #9 observed that despite mentioning their dietary preferences as being a vegetarian in the user profile, the agent recommended options which did not conform with that. Participant #14 found that the chatbot was also not able to justify its choices clearly when making recommendations. Multiple participants also commented on the inability of the GPT to provide complete information in its response. For instance, when recommending product names, it often forgot to mention the price and location, which had to be requested for separately.

Overall, although the GPT possesses its knowledge retrieval functionality, the efficiency of the same is reduced when the number of items to be retrieved is higher. This leads to either hallucinations or omissions, both of which are detrimental in the case of a supermarket chatbot, as a hallucination misleads the user into believing that certain products which do not exist are available, while omissions can lead to lost opportunities to recommend appropriate items for the customer.

#### 6.4.3 Benefits of custom multi-LLM chatbot

Participants overall agreed that the proposed chatbot was direct and efficient. Multiple participants commented on the preciseness of the answers, which they found made the chatbot very helpful. Although participants were asked to only evaluate the chatbot based on the responses, participants were also impressed with the speed of the chatbot. Participant #4 commented on how the chatbot reminded them of certain ingredients for their dish that they had forgotten, which was very useful. Participant #5 mentioned that they found the ability to ask questions to narrow down the options to be a helpful feature in the agent. Participant #7 commented about the reliability and trustworthiness of the agent on account of both the format and reasoning provided by the chatbot. Participant #11 also mentioned how this chatbot could be useful for people who tend to be more socially anxious and wary of approaching the workers in the supermarket for help and recommendations.

The multi-LLM approach is more to the point on account of being fine-tuned on task-related conversations. By using multiple smaller models, inference speed is greatly increased compared to the GPT4 alternative, whilst also reducing costs. The usage of our own retrieval system proved to be more effective than the alternative used internally by the GPT’s knowledge retrieval functionality. The ability of the high-level LLM to break down and list all the potential items needed for a complex query ensured the customer never forgot about any item which was perceived as useful. Since the medium-level and low-level LLMs were fine-tuned on providing reasoning in their responses, the overall credibility of the system was improved as well.

#### 6.4.4 Concerns regarding the custom multi-LLM chatbot

In general, participants felt that the chatbot’s ability to provide detailed recipes, ideas or plans outside the scope of product recommendation was fairly limited. Participant #2 stated that they felt the chatbot was more coercive and ‘pushy’ by trying to force them towards specific products. Participants #3 and #5 found that the chatbot made errors when summarising the final list or maintaining track of the conversation. Participant #8 found that when the LLM was asked to provide the total price of all products, the answer was incorrect. Participant #13 also commented on how the tool may lead to them purchasing more than they initially sought out.

One of the main issues with the multi-LLM approach was when the query of the user was misclassified. Thus, when a high-level query was misclassified as low-level, or *vice versa*, typically unsatisfactory results were obtained. This is primarily caused due to the inability of either approach to respond to queries that aren’t in line with the strategies employed by both approaches. For instance, if a high-level query is misclassified as low, the information retrieval is fairly poor and relevant items are not extracted from the database. Meanwhile, if a low-level query is classified as high-level, the necessary context of the previous conversation is not available, leading to confusion in terms of recommendations by the agent.

## 7 Integration into robotics

The conversational agent is a powerful tool to help customers in a supermarket find what they are looking for, get useful information and also obtain personalised recommendations based on their preferences. While this chatbot can be applied as a standalone application on a mobile phone or kiosk at the entry of the supermarket, we are also interested in exploring how these chatbots can be effectively integrated into high-level robot planning to guide a supermarket robot to go to the necessary locations after which the required low-level perception, motion planning of a manipulator and control can be applied for automated object retrieval and collection. This feature is useful as it can allow a customer to interact with the chatbot and have a robot autonomously pick up the necessary items and bring them to the user. While the low-level functionality, such as perception and manipulation, is beyond the scope of our work, we demonstrate with a simple example how our robot can navigate to the necessary shelves after receiving an appropriate request from the customer.

The key assumption made in this work is that the position of all shelves remains the same over time. This is a reasonable assumption to make since most path-planning algorithms require a pre-recorded map to facilitate path planning from a given start point to a destination. If the supermarket is to change its overall configuration, a new map would have to be generated by using SLAM or other similar mapping techniques.

To connect the chatbot with the robot, we use an LLM to process the final conversational agent message, which has a list of all the products the customer has indicated a willingness to purchase and retrieve a list of shelf numbers for each object. The prompt for this LLM is provided in Supplementary Appendix SAG.4. We then define this as a set, removing any duplicates in case multiple items are on the same shelf. The shelves can then be arranged to optimise the total distance covered by the robot. We then look up the specific shelf numbers position from a pre-configured YAML file consisting of the X-Y coordinates of the shelves to retrieve the destination and end pose of the robot. We iterate over all the shelves one after the other until the robot has visited all the necessary items.

**FIGURE 6:**
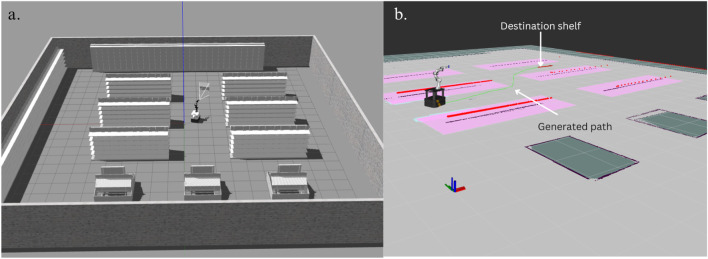
The robot in a large simulated supermarket. **(a)** Shows the render on Gazebo while **(b)** shows the path (in green line) and the robot navigating to the correct shelf in RViz. The simulation and demonstration have been done on ROS Noetic.

For the simulation shown in Figure 6, we built on ROS Noetic using a Clearpath mobile base robot with a Franka Emika arm. The local planner for the mobile base was set as Timed Elastic Band (TEB) Rösmann et al. (2012) and localisation as Adaptive Monte Carlo Localisation (AMCL) Chung and Lin (2022). After reaching the shelf, the necessary perception, motion planning and control nodes can be called so as to facilitate picking up the right object and adding it to the supermarket basket. Once this is done, the next shelf can be visited and so on until all items are retrieved. The robot can then navigate to the checkout to deliver the items to the customer. This is a simple yet effective manner in which our solution can be integrated into a supermarket robot.

## 8 Discussion

After presenting our approaches and results for building equitable and effective agents, we now discuss the key learnings and takeaways along with limitations and scope for future work.

### 8.1 Discussion on language and gender parity

Based on the results in Section III, we make multiple observations. Firstly, OpenAI’s Whisper has proven to be the best speech recognition system by being significantly better than its peers. It also retains this accuracy for Dutch and different genders. Microsoft Azure Speech comes next, followed by Google Cloud Speech. Vosk, the other open-source model, can run locally and has a fast inference time, albeit at a higher error rate.

Whisper also has other benefits that make it the clear winner in this evaluation:

1. Whisper is open source - allowing it to be run locally, given the necessary hardware requirements and can be fine-tuned with more speech data for specialised purposes, improving its performance. While both Microsoft and Google allow training on the consoles as well, data privacy concerns and costs may make them less attractive than Whisper.
2. Unlike the other models, for the evaluation, it was observed that Whisper was the only model that was capable of recognizing the language by itself, while other models needed the language passed as a parameter or in the case of Vosk, to add the path to the files needed for the specific language.
3. Remarkable developments are still being made to improve the speed of inference of Whisper, allowing for faster versions of the same created by the community, which could help reduce latency.

We also notice that the Word Error Rate for the same models is higher in the Dutch language than in English. This is to be expected since most of the models have been trained on far longer durations and greater quantities of data in English rather than Dutch. However, if we intend to build systems that can be deployed to the general public in the Netherlands, efforts must be made to fine-tune these open-source models on large amounts of Dutch audio-transcription data so that higher accuracy can be achieved.

The limitation of the current study is a small sample size, preventing deeper analysis into other factors such as the accent and age of the speaker–important variables in the performance of speech recognition systems. Rajan et al. (2022) states that Nigerian women, for instance, have significantly higher error rates than white caucasian males. Analysing the accent of the person could provide further insights into the robustness of such systems and if they have certain biases that can be rectified by fine-tuning representational data, which also includes the marginalized group. Furthermore, using other metrics like Word Information Lost and Match Error Rate could be used to further test our results and get better insights into the performance of these models. Furthermore, we have done analysis of only two languages and two genders. This is mainly attributed to the desire to build systems that cater to Dutch supermarkets, where the study was based out of. We note this as a limitation and would like to urge future work to collect larger data samples across multiple languages and genders, whilst also accounting for accent and demographics.

### 8.2 Discussion on multi-LLM conversational agent

Overall, we observe that the multi-LLM approach offers multiple benefits over using the most powerful LLM, like the state-of-the-art GPT, such as reduced costs, reduced latency, increased control over specialised tasks, easier ablation and comparative studies and better task performance. While the GPT solution is indeed the quickest and easiest in terms of deployability, the performance of knowledge retrieval is rather inadequate. By utilising multiple smaller LLMs capable of interacting with one another and maintaining a common conversation log helps in providing context to each separate model as well. The presence of a classifier enables us to directly route queries to the correct model instead of following a common approach for all questions. By fine-tuning with GPT4 augmented data we are also able to leverage the formatting and style of the responses to be extremely well structured and easy to understand.

Furthermore, the modular nature of our solution enables easy substitution of models with alternatives as they become available making the solution extremely flexible to adapt to future developments in the field. One can also fine-tune and use open-source models to ensure reliability and address concerns regarding data privacy and security. Furthermore, by increasing or optimising the number of classes the classifier can select items into, other roles can also be unlocked, such as bill management, asking for assistance from supermarket workers or providing feedback. The approach is also not limited to supermarket scenarios and can be easily applied to other domains which could benefit from utilising voice-based interfaces. By selecting the type of LLMs and queries, the approach can be optimised based on the specific task. For example, if one were to build a polishing robot for the industry, aspects such as parity and effectiveness would still be significant. Furthermore, instead of having 4 classes of queries, the number could be reduced to 2 control queries, where the user specifically sets values, such as impedance or high-level generic queries, such as outlining the overall task.

However, the current approach is not without its share of limitations. Incorrectly classified queries can lead to the query being handled by a model that is not specialised for the given task. This could potentially lead to a loss of context and confusing results to users. Since the classifier is built atop a multilingual BERT classifier, the responses are highly sensitive to changes in spellings and the manner in which the customer expresses themselves. We believe replacing the mdistilBERT classifier with a small fine-tuned LLM tasked with query classification and rewriting to add any necessary context could be a viable solution to address these limitations and add context to a query to improve retrieval. Next, the number of examples used to fine-tune the different models is relatively lower (30–50) and future efforts must attempt to increase the number of examples to obtain better results. The extra examples should also involve usage of Dutch conversations. Although Large Language Models are pre-trained to be well-versed in multiple languages, providing extra examples in other relevant languages can ensure representational fine-tuning that can also pick-up cultural norms, common sayings and conversational habits native to the language. Lastly, we believe greater research and insights need to be uncovered about ensuring the scalability of the current system. While the runtime API costs have been covered in Section 5, other factors such as computational resources, average response time per model and real-world implementation challenges must also be covered in future studies. These factors are difficult to uncover with closed-source models as exact model size and parameter count are not publicly available. Furthermore, response time is heavily based on query length, type of model called (for example, the high-level LLM provides shorter responses as it asks questions to the user while the mid-level LLM provides detailed responses), server load and other variables. Selecting or defining good metrics to measure the same can help quantify the degree of scalability of these systems in the long term.

## 9 Conclusion

This thesis advances research in the field of voice-based interface research for supermarket applications and robots, emphasising equitability and the development of a novel multi-LLM agent. In terms of equitability, our findings show that OpenAI’s Whisper outshines three other leading speech recognition systems in robustness and accuracy. Furthermore, our findings reveal a higher Word Error Rate for Dutch than English, which underscores the necessity for specialized ASR systems in Dutch settings. Our multi-LLM agent surpassed the state-of-the-art in 4 of 13 parameters and demonstrated better performance across all 13 measured ASAQ criteria. The successful integration of LLMs into robot path planning for shelf-directed item retrieval exemplifies the practical application of these interfaces in real-world settings. These studies and experiments set the stage for developing more equitable, customized, and effective interfaces across various domains.

## Data Availability

The original contributions presented in the study are included in the article/Supplementary Material, further inquiries can be directed to the corresponding authors.
