# Supplementary material for: Enhancing supermarket robot interaction: an equitable multi-level LLM conversational interface for handling diverse customer intents
Source: Front Robot AI. 2025 Apr 29;12:1576348. doi: 10.3389/frobt.2025.1576348 (PMC12069059; doi:10.3389/frobt.2025.1576348)
Supplement: Supplementary file 1 [file DataSheet1.pdf]

frontiers

Supplementary Material

1 PARTICIPANT INSTRUCTIONS

For all three experiments, clear instructions were provided to the participants on how to contribute effectively to the study. Below, we outline the instructions for each experiment.

1.1 Speech Recognition Experiment

Participants were welcomed and thanked for participating in the study. Afterward, the informed consent form and details about the experiment and data privacy were communicated. Participants were categorized into one of four groups: Dutch Male, Dutch Female, English Male, and English Female. Each group was exclusive to prevent any interference by allowing the same participant to speak in multiple languages.

The FiFine microphone was set up and tested on Audacity before every recording, ensuring the experiment was conducted in a low-noise environment. Participants were shown the script they would read and were given as much time as they needed before dictating the text. Participants were asked to speak both the lines of the user and chatbot into the microphone, and the recording was captured via Audacity. For all 40 participants, the microphone configuration and settings on Audacity were maintained at the same default level. If participants made a mistake, they were allowed to re-record the audio file.

After recording, the audio file was tested to ensure there were no errors in audio capture. The audio file was saved as an MP3 and given a unique ID to link it to the participant. In a separate file with the same ID name, the gender and accent of the participant were recorded.

1.2 Personalization Experiment

Participants were welcomed and thanked for participating in the study. Afterward, the informed consent form and details about the experiment and data privacy were communicated. Participants were placed into two groups based on the order in which they interacted with the control/personalized chatbots. The order was alternated for each subsequent participant. Participants were informed that they would be interacting with two chatbots—one after the other—and that they would have to choose which chatbot they preferred more at the end of the experiment.

Participants began by filling out the onboarding questionnaire in both cases, obtaining a QR code that served as their membership card, and then interacting with the chatbot for a maximum duration of five minutes. After this, participants were asked to repeat the same procedure for the other chatbot. The onboarding and QR code step was used even for the non-personalized chatbot to mitigate any potential biases, but the retrieved information was not used in the control agent. At the end of the experiment, participants were asked to record which chatbot—the first or the second—they preferred more.

1.3 Interaction with Novel Multi-LLM Agent vs. State-of-the-Art GPT

Participants were welcomed and thanked for participating in the study. Afterward, the informed consent form and details about the experiment and data privacy were communicated. Participants were placed into two groups based on the order in which they interacted with the GPT/multi-LLM chatbots. The order was alternated for each subsequent participant. Participants were informed that they would be interacting with two chatbots—one after the other. The overall experiment took roughly 40 minutes per participant.

Before beginning, required demographic information was collected, such as age, gender, familiarity, and frequency of usage of LLMs, to ensure that all participants had interacted with such agents to prevent any learning effects from interfering with the study. Next, participants were asked to reflect on their general intentions during a supermarket visit. The specific instruction provided was:

Please describe, in around 100 words, any objectives or inquiries you might have while visiting a supermarket. This could range from searching for particular items, seeking advice or recommendations, to any general queries you often find yourself pondering amidst the aisles. Feel free to reflect on your personal needs, preferences, or a specific list of items you aim to purchase.

A useful way to approach this is to think about the types of products or goods that usually draw your interest, or those you suddenly remember you need once you're there. Your input can draw upon both past shopping experiences or current needs.

This question was asked to help participants mentally prepare themselves by thinking about what they would potentially look for in a supermarket so that they could interact with the chatbot more naturally. After this, they were provided with the first chatbot and asked to interact with it until they felt satisfied with the results or had enough to make an assessment. They were then asked to fill out the relevant questions in the ASAQ questionnaire. Following this, three qualitative questions were provided to which the participants could write their views in whatever depth they deemed necessary. The same process was then repeated for the other chatbot.

2 SCRIPT FOR EVALUATION OF SPEECH RECOGNITION SYSTEMS

Participants were asked to read both lines without saying ”user” or ”chatbot.” The script was created based on a common interaction in a supermarket and consists of multiple words and phrases that are commonly utilized.

2.1 English Version

”Hello, I'm planning a big family dinner and need to do some shopping. Can you help me find a few items?”
”Of course! What are you looking for?”
”Let's start with fresh produce. I need tomatoes, lettuce, and cucumbers for a salad.”
”You can find those in the fresh produce section, aisle 4.”
”Great, thanks. I also need to pick up some seafood. Do you have salmon and shrimp?”
”Yes, our seafood selection is in aisle 9. You'll find both salmon and shrimp there.”
”Perfect. I'm thinking of making a dessert too. Where can I find chocolate chips and vanilla extract?”
”For baking supplies, head over to aisle 7. You'll find chocolate chips and vanilla extract there.”
”Also, I almost forgot, where's the pasta section?”
”Pasta is in aisle 6, right next to the Italian sauces.” ”Thank you. That should be all for now.”
”You're welcome! Let me know if there's anything else I can assist with. Have a great shopping experience!”
”Actually, just one more thing. Where can I find cleaning supplies?”
”Cleaning supplies are located in aisle 11. Is there a specific brand you're looking for?”
”No, that's all. Thanks again!”
”No problem at all. Goodbye and take care!”

2.2 Dutch Version

”Hallo, ik plan een groot familiediner en moet wat boodschappen doen. Kun je me helpen een paar artikelen te vinden?”
”Natuurlijk! Waar bent u naar op zoek?”
”Laten we beginnen met verse producten. Ik heb tomaten, sla en komkommers nodig voor een salade.”
”Die vindt u in de versafdeling, gang 4.”
”Geweldig, bedankt. Ik moet ook wat zeevruchten halen. Hebben jullie zalm en garnalen?”
”Ja, onze selectie aan zeevruchten bevindt zich in gang 9. Daar vindt u zowel zalm als garnalen.”
”Perfect. Ik denk erover om ook een dessert te maken. Waar kan ik chocoladestukjes en vanille-aroma vinden?”
”Voor bakbenodigdheden kunt u naar gang 7 gaan. Daar vindt u chocoladeschilfers en vanille-extract.”
”O ja, ik was bijna de pasta vergeten. Waar ligt de pasta?”
”Pasta vindt u in gang 6, naast de Italiaanse sauzen.”
”Dank je. Dat zou alles moeten zijn voor nu.”
”Graag gedaan! Laat het me weten als ik nog ergens anders mee kan helpen. Een prettige winkelervaring toegewenst!”
”Eigenlijk, nog één ding. Waar kan ik schoonmaakartikelen vinden?”
”Schoonmaakartikelen vindt u in gang 11. Zoekt u een specifiek merk?”
”Nee, dat is alles. Nogmaals bedankt!”
”Geen probleem. Tot ziens en zorg goed voor uzelf!”

3 INFORMED CONSENT - SPEECH RECOGNITION EXPERIMENT

Research Study Invitation:

You are being invited to participate in a research study titled *Evaluating Off-the-Shelf Automated Speech Recognition Models for Voice-based Control of Robots: A Comparative Study*.

This study is being conducted by Chandran Nandkumar, a Master's student from TU Delft, under the guidance of Dr. Luka Peternel for the scope of his Master thesis.

The purpose of this research study is to compare different commercially available Speech recognition systems for different genders and languages (English and Dutch), and will take you approximately 15-20 minutes to complete. The data will be used for evaluating which models of speech recognition are the most robust to handle different types of users and their potential use for voice-based control of robots. We will be asking you to provide certain scripted voice commands to a robot in a supermarket setting along with data regarding your personal information such as gender, accent, and nationality for analysis of the results.

As with any online activity, the risk of a breach is always possible. To the best of our ability, your answers in this study will remain confidential. We will minimize any risks by separating contact information from personal information, deleting the data after analysis and verification of results, and taking all necessary steps to ensure data is protected by suitable encryption and using OneDrive via the TU Delft account to store details.

Your participation in this study is entirely voluntary, and you can withdraw at any time. You are free to omit any questions.

TEMPLATE 2: Explicit Consent points

Please make sure that you select (and amend as necessary) any Explicit Consent points which are relevant to your study and exclude those which do not apply. You should also add further points and necessary to address your specific research situation.

PLEASE TICK THE APPROPRIATE BOXES	Yes	No
**A: GENERAL AGREEMENT – RESEARCH GOALS, PARTICPANT TASKS AND VOLUNTARY PARTICIPATION**		
1. I have read and understood the study information dated 12/12/2023 or it has been read to me. I have been able to ask questions about the study and my questions have been answered to my satisfaction.	☐	☐
2. I consent voluntarily to be a participant in this study and understand that I can refuse to answer questions and I can withdraw from the study at any time, without having to give a reason.	☐	☐
3. I understand that taking part in the study involves: recording audio of specific voice commands given to a supermarket robot whose script has been handed over to you. The data will be recorded in mp3 format and information regarding your gender, accent and language will be used to compare the performance of the different speech transcription models. The recording provided will be destroyed after the completion of the analysis and latest by the end of May 31st, 2024.	☐	☐
4. I understand that the study will end before May 31st, 2024 (latest) and all the data collected will be destroyed.		
**B: POTENTIAL RISKS OF PARTICIPATING (INCLUDING DATA PROTECTION)**		
5. I understand that taking part in the study involves the following risks – reading out certain non-offensive, fixed script commands to a supermarket robot which may be mildly discomfortable for certain long phrases and commands; furthermore – multiple recordings may be requested due to technical difficulties, need to increase sample size and capture the best possible voice recording. I understand that these will be mitigated by allowing the participants to take breaks whenever they would like, providing water and/or any refreshment that may help them with participating in the study.	☐	☐
6. I understand that taking part in the study also involves collecting specific personally identifiable information (PII) such as name, gender, nationality, accent and associated personally identifiable research data (PIRD) of the voice recordings for certain given voice commands with the potential risk of my identity being revealed indirectly after data analysis via a publication in a Master thesis, or a conference/journal publication	☐	☐
7. I understand that some of this PIRD is considered as sensitive data within GDPR legislation, specifically - None	☐	☐
8. I understand that the following steps will be taken to minimise the threat of a data breach, and protect my identity in the event of such a breach -<br>a. All information will be stored in OneDrive via the TU Delft account and the personal data will be configured as online only files ensuring that there is no access to the data on any local computer<br>b. The voice recordings may also be used via the personal computer but encryption standard like using BitLocker and regular updates will be performed to ensure that the safety is ensured.<br>c. The contact details of the participant such as name, number, email will be separate from there PII using a unique random ID number linking the two. This ensures the personal information cannot be traced back to the participant.	☐	☐

PLEASE TICK THE APPROPRIATE BOXES	Yes	No
d. All data will be destroyed promptly after analysis or publication and verification of results latest by May 31st , 2024.		
9. I understand that personal information collected about me that can identify me, such as name, email id, contact number, gender, nationality, accent and voice recordings will not be shared beyond the study team.	☐	☐
10. I understand that the (identifiable) personal data I provide will be destroyed promptly after analysis or publication and verification of results latest by May 31st , 2024.	☐	☐
**C: RESEARCH PUBLICATION, DISSEMINATION AND APPLICATION**		
11. I understand that after the research study the de-identified information I provide will be used for publication in the results for my master thesis and/or potential publication in a journal or conference. The objective of such a study would be to understand how speech transcription models are capable of handling different genders, languages, nationalities, accents and which transcription model is the best for giving voice commands to robots in a supermarket setting.	☐	☐
12. I agree that my responses, views or other input can be quoted anonymously in research outputs	☐	☐
**D: (LONGTERM) DATA STORAGE, ACCESS AND REUSE**		
13. I give permission for the de-identified contact information, personal information and voice recordings that I provide to be archived in OneDrive via TU Delft account repository so it can be used for future research and learning.	☐	☐
14. I understand that access to this repository is restricted only to the principal investigator of the study. The personal and contact information will be stored in online only mode while the voice recording will be stored on a personal computer which is encrypted by BitLocker and will be regularly updated to abide by the latest security protocols. The data will be deleted after analysis or verification of the results latest by 31st May 2024	☐	☐

Signatures

______________________________	____________________________	_________
Name of participant [printed]	Signature	Date

4 NORMALITY TESTS, PAIRWISE T-TESTS OF ALL GROUPS FOR ALL MODELS

We begin by checking all the groups for normality using the Shapiro-Wilk test. We have 4 groups: Dutch Female, English Female, Dutch Male, and English Male. We also have 4 models: Microsoft Azure, Google Cloud Speech to Text, OpenAI Whisper and Vosk. Thus, there are 16 different configurations for which we apply the Shapiro-Wilk test. The same has been tabulated in S1.

Table S1. Shapiro-Wilk Tests for Normality of WER Scores

Group	Model	Statistic	P-Value
Dutch Female	Microsoft	0.890	0.169
Dutch Female	Vosk	0.879	0.127
Dutch Female	Google	0.912	0.298
Dutch Female	Whisper	0.912	0.293
English Male	Microsoft	0.913	0.303
English Male	Vosk	0.939	0.546
English Male	Google	0.947	0.634
English Male	Whisper	0.889	0.165
Dutch Male	Microsoft	0.973	0.920
Dutch Male	Vosk	0.958	0.765
Dutch Male	Google	0.943	0.588
Dutch Male	Whisper	0.971	0.898
English Female	Microsoft	0.661	0.000298
English Female	Vosk	0.907	0.258
English Female	Google	0.890	0.170
English Female	Whisper	0.893	0.182

As we can see, only one of the combinations (English Female Microsoft) fails the Shapiro-Wilk test and thus deviates from the normal distribution. We thus perform the Wilcoxon-signed-rank test for English female Microsoft and other models while using the parametric t-test for the rest.

5 DUTCH FEMALE

Model 1	Model 2	T-Statistic	P-Value
Microsoft	Vosk	-3.49	0.0026
Microsoft	Google	-2.10	0.0501
Microsoft	Whisper	1.70	0.1055
Vosk	Google	0.84	0.4139
Vosk	Whisper	5.41	0.000038
Google	Whisper	3.43	0.0030

Table S2. Pairwise T-Tests for Dutch Female Group

6 ENGLISH FEMALE

Model 1	Model 2	T-Statistic	P-Value
Vosk	Google	1.28	0.2185
Vosk	Whisper	7.16	0.0000011
Google	Whisper	4.79	0.000146

Table S3. Pairwise T-Tests for English Female Group (no Microsoft as it violated the Shapiro-Wilk test)

Table S4. Wilcoxon Signed-Rank Tests for English Female Group Comparisons with Microsoft

Comparison	Statistic	P-Value
Microsoft vs Whisper	10.0	0.1386
Microsoft vs Google	0.0	0.00195
Microsoft vs Vosk	0.0	0.00195

7 DUTCH MALE

Model 1	Model 2	T-Statistic	P-Value
Microsoft	Vosk	-4.44	0.00032
Microsoft	Google	-3.30	0.0039
Microsoft	Whisper	1.71	0.1046
Vosk	Google	1.67	0.1117
Vosk	Whisper	6.73	0.0000026
Google	Whisper	6.22	0.0000071

Table S5. Pairwise T-Tests for Dutch Male Group

8 ENGLISH MALE

Model 1	Model 2	T-Statistic	P-Value
Microsoft	Vosk	-7.71	0.000000414
Microsoft	Google	-4.79	0.000147
Microsoft	Whisper	1.21	0.2423
Vosk	Google	2.26	0.0368
Vosk	Whisper	8.11	0.000000200
Google	Whisper	5.29	0.000050

Table S6. Pairwise T-Tests for English Male Group

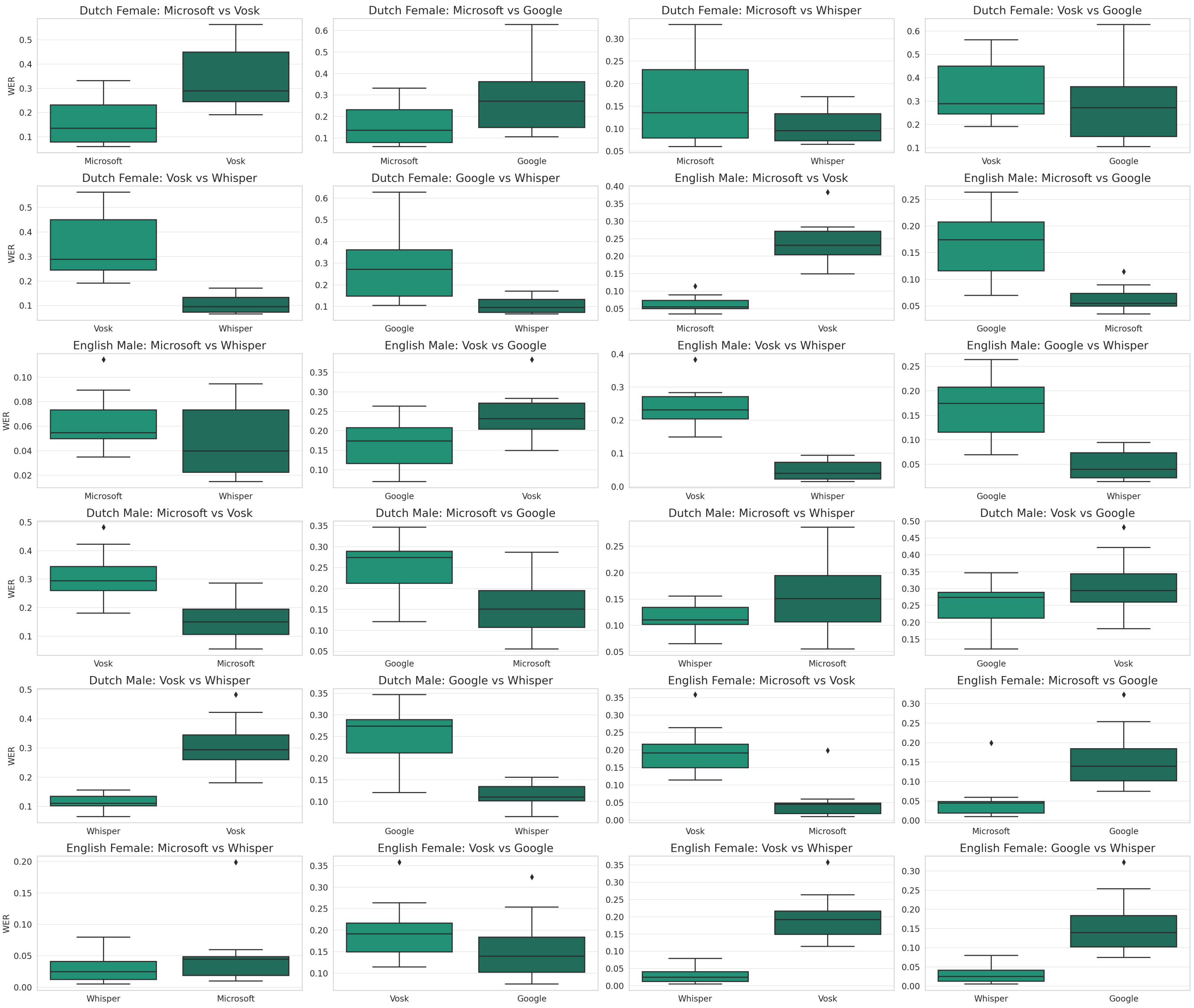

Figure S1. All the pairwise box plots of 4 models for all 4 groups.

We thus note that Whisper by OpenAI is significantly better than Google Cloud Speech to Text and Vosk for all 4 groups. However, there is no statistical significance that Whisper is better than Microsoft Azure speech for any of the groups. This is also fairly evident looking at the boxplots in Fig. S1.

However, when we combine all groups and then perform the t-test, we find that Whisper is significantly better than even Microsoft, albeit to a lesser degree than Google and Vosk, as seen in Table S7.

Table S7. Pairwise T-Tests for Models Across All Groups

Model 1	Model 2	T-Statistic	P-Value
Microsoft	Vosk	-7.84	1.88e-11
Microsoft	Google	-5.02	3.19e-06
Microsoft	Whisper	2.19	0.0317
Vosk	Google	2.31	0.0236
Vosk	Whisper	10.84	3.21e-17
Google	Whisper	7.44	1.15e-10

9 EFFECT OF PERSONALISATION AND RESULTS

This experiment was done as a part of a course at TU Delft titled Conversational Agents (CS4270) along with 3 other teammates. While the complete experiment covered a greater number of aspects, this appendix lists only the major contributions of the author. However, since participant recruitment was done as a joint effort, this experiment has been covered in the Appendix.

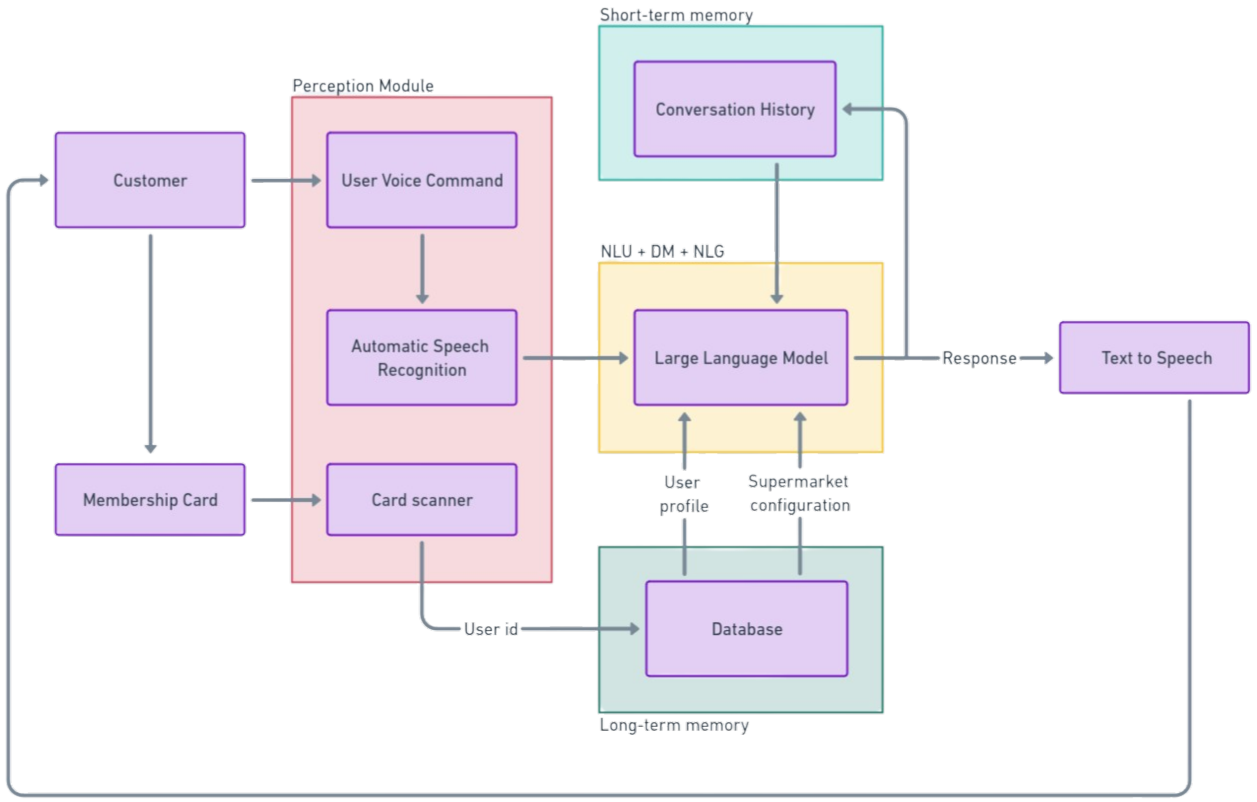

Figure S2. Conversational Agent Architecture

9.1 Components of the basic conversational agent

Now that we have a clear direction of how to achieve personalisation based on the classification by supermarket retail salespeople (Section IV), we must set up a simple end-to-end architecture to facilitate communication between the customer and the agent. We thus organise the different elements of the agent as follows -

9.1.1 Memory

The information about the user's personal preferences is recalled after scanning their membership card. This information, after collection during customer onboarding, is stored as a user profile in a database. We use MongoDB, a noSQL database, for this purpose due to its popularity and reliability. This information is provided to the chatbot to help guide its decision making.

9.1.2 Perception

Our conversational agent leverages ASR and QR code detection technology as input to improve the grocery shopping experience for the customer. With regards to the component of user identification, we believe that using a QR code scanner holds the highest promise due to: a) reduced privacy risk compared to methods such as facial recognition, b) lower cost of deployment, and c) lower latency. Furthermore, by using the speech recognition system, we can convert the customers' instructions into plain text, which can be written in multiple languages, easily modified and trained upon, offers low latency, and offers the most opportunities to prevent Personally Identifiable Information (PII) from being leaked.

Thus, we use a camera and a microphone as our perception modalities. The camera is used to scan the QR code and the microphone enables the conversion of speech in any of the supported languages into text by employing Whisper by OpenAI. Together, these should be able to retrieve user information and receive user input to enable conversations with the user.

9.1.3 Natural Language Understanding, Dialogue Management and Natural Language Generation

With regards to the selection of the natural language understanding, dialogue management and natural language generation module, we opt to use a single powerful LLM. We chose to use the GPT-4-Turbo model due to its higher context window, advanced reasoning abilities and its ability to retain relevant context cues and user information, but suffered from significant latency, which makes response time in certain cases rather long.

9.2 Experiment setup and methodology

In order to evaluate the effectiveness of our personalised chatbot compared to a non-personalised control chatbot with identical architecture, we implemented a blindfolded, counterbalanced study. This study aimed to determine if the addition of user-specific information in chatbot responses and decision-making processes is favored over the use of a generic, memory-less control chatbot. Participants were split into two groups: one group first engaged with the non-personalised chatbot and then with the personalised chatbot, while the second group had the reverse order of interaction. All participants were kept unaware of which version of the chatbot they were interacting with at any time, minimizing potential bias or pre-existing opinions that could influence their experience. This method ensured that every participant had the opportunity to interact with both versions of the chatbot, effectively eliminating any order-related bias in the process.

For this experiment, 30 random participants were selected. 22 participants were male and 8 participants were female. This experiment alone was performed with the assistance of three other students as a part of a course. The age of the participants was not recorded, but they were recruited from the university campus. Participants are asked at the end of the experiment which chatbot they preferred–the first or the second.

The participants will be informed that they will be interacting with two chatbots for a period of 5 minutes each to achieve their goals during a typical visit to the supermarket. No other information about the differences between the chatbots are provided. In both cases, they create an account by filling in the initial onboarding questions, are presented with a QR code that serves as their membership card and lastly are asked to scan the QR code to use the chatbot.

Participants are presented with both chatbots, one after the other. The order in which the control and personalised agent are presented is cycled. Efforts are made to ensure there is no means of understanding which chatbot is personalised and which isn't. Finally, the participants are asked which of the two chatbots they prefer. For our study, we will be employing the Chi-square test of independence for the user preference.

Since all participants have been asked to pick the chatbot they prefer, we will be able to determine if there is a statistically significant preference for either chatbot. This will ensure that we can base our results not solely on the total number of participants who preferred with vs without memory, but even account for the randomness induced by differences in experiment order.

9.3 Results of the evaluation of our proposed personalisation of responses vs default LLM

The final preferences of all participants are in Table S8. Overall, 21 of the 30 participants preferred the chatbot with memory while 9 preferred without. To determine if this is significant we perform a chi-squared test for independence. The analysis yielded a chi-squared statistic of $\chi^2(1, N = 30) = 5, p = .025347$ making our experiment statistically significant (p = 0.025, α = 0.05). These findings indicate that there is a significant association between the incorporation of personalisation into a chatbot and user preference.

Table S8. User Preferences for Chatbots with and without personalisation. Group 1 comprises of all those participants who first tried the model with personalisation and then without, while Group 2 comprises of all participants who first tried the chatbot without personalisation and then with.

	First chatbot	Second chatbot
**Group 1**	9	6
**Group 2**	3	12

10 RELEVANT ASAQ QUESTIONS AND THE RELEVANT CRITERIA

For the evaluation of our custom multi-LLM chatbot with the state-of-the-art, we used the Artificial Social Agent Questionnaire (ASAQ). The questionnaire comes in two formats: a long version and a short version. Regardless of the length, the ASAQ provides insights into 19 criteria for an artificial social agent as seen in Table S9.

Given the nature of our study, the long version of the ASAQ was impractical as it comprised of 90 questions. Asking participants to respond to all 90 questions–twice is not only infeasible in terms of time, but could also discourage participation. Furthermore, some criteria in the ASAQ were not deemed relevant for our study. These criteria include–believability (Does the ASA resemble human or a nature being? Is it suitable for its role?), sociability (Can the ASA easily interact with the user socially?), Social Presence

Table S9. Criteria and Their Conveyance in ASA Evaluation

Sl. No.	Criteria	What It Conveys
1	Believability	Does the ASA resemble a human or a natural being? Is it suitable for its role?
2	Usability	Is the ASA easy to use and to learn?
3	Performance	Does the ASA accomplish its task?
4	Likeability	Does the user like the ASA? Is it pleasing?
5	Sociability	Can the ASA easily interact with the user socially?
6	Personality Presence	Does the ASA have a distinctive character?
7	User Acceptance	Does the user intend to interact with the ASA again in the future?
8	Enjoyability	Does the user enjoy interacting with the ASA?
9	User Engagement	Did the interaction capture the user's attention?
10	User Trust	Does the ASA always give good advice? Is it trustworthy and reliable?
11	User-Agent Alliance	Do the ASA and the user have a strategic alliance?
12	Attentiveness	Is the ASA attentive?
13	Coherence	Does the ASA's behavior make sense?
14	Intentionality	Does the ASA have a clue of what it is doing?
15	User Attitude	Does the user see the interaction with the ASA as something positive?
16	Social Presence	Does the ASA have a social presence?
17	Impact on Self Image	Would others (who are close to the user) encourage the user to use the ASA?
18	Emotional Experience	Can the ASA express its emotion? Are the user's emotions caused by the ASA?
19	User-Agent Interplay	Do the ASA's and the user's emotions affect each other?

(Does the ASA have a social presence?) Emotional Experience (Can the ASA express its emotion? Are the user's emotions caused by the ASA?) and User-Agent Interplay (Do the ASA's and the user's emotions affect each other?). This leaves us with the following 13 questions and 13 criteria.

Table S10. Mapping of ASAQ Questionnaire Questions to Criteria

Sl. No.	Question	Criteria
1	The agent is easy to use.	Agent's Usability
2	The agent does its task well.	Agent's Performance
3	I like the agent.	Agent's Likeability
4	I will use the agent again in the future.	User Acceptance of the Agent
5	The agent is boring.	Agent's Enjoyability
6	The interaction captured my attention.	User's Engagement
7	I can rely on the agent.	User's Trust
8	The agent and I have a strategic alliance.	User-Agent Alliance
9	The agent is attentive.	Agent's Attentiveness
10	The agent's behavior does not make sense	Agent's Coherence
11	The agent has no clue of what it is doing.	Agent's Intentionality
12	I see the interaction with the agent as something positive.	Agent's Attitude
13	Others would encourage me to use the agent.	Interaction Impact on Self-Image

11 PROMPTS PROVIDED TO ALL THE LLMS

11.1 High-Level LLM

The prompt presented below is used to instruct the high-level LLM to interact with the user and break down the high-level request into smaller discrete components. The only variable input in this case is the user profile information appended as a string in the second line. The last two lines are added in uppercase to increase emphasis and ensure the LLM follows them.

System Prompts:

You are a supermarket high-level chatbot tasked with taking a user's query and asking them for additional information needed to correctly satisfy their request by understanding their needs and intentions.

This is the user profile that gives you a general picture of the user's nature - + ¡customer profile information¿

Based on your interaction, you will create a list of items and seek feedback on the created list from the customer. When you detect that the customer is happy with the list you have created, you will give your final response starting with ¡End-Of-Conversation¿ followed by the comma-separated list inside .

You need to ensure the items in the list are not too specific nor too general–unless the user has specified the same.

Your final response will be fed to a low level function which will retrieve the information from the database of the supermarket–so ensure you ignore all the low level aspects of the query like price, discounts, quantity and brand; that is the job of the low level function–you need to only worry about generating the necessary items. So ensure the format is good. Be kind respectful and helpful to the user.

REMEMBER THAT YOUR CONVERSATION AND RECOMMENDATIONS ARE PRETTY GENERIC WITH SOME SMALL SPECIFICITY. ALSO ALWAYS SAY THAT YOU WILL LOOK FOR THE ITEMS AND NOT THAT YOU HAVE THOSE ITEMS.

LASTLY, ALWAYS REMEMBER TO ASK FOR USE CONFIRMATION BY REPEATING THE LIST BACK TO THEM AFTER EACH CHANGE. WHEN YOU FEEL THEY ARE HAPPY, SAY End-Of-Conversation FOLLOWED BY THE COMMA-SEPARATED LIST INSIDE

11.2 Medium-Level LLM

The prompt presented below is used to instruct the medium-level LLM to process the output of the high-level LLM and output in a human-readable and friendly manner the list of relevant products. The variables that are provided as input in this case are the retrieved context comprising of relevant items, the chatlog with the high-level LLM for context of what the user preferred and what they did not, the output of the high-level LLM and the user profile information added as the user input.

System Prompts:

You are a mid-level assistant supermarket chatbot that serves the request of a high-level chatbot. You take 4 inputs: 1. a large amount of context generated by the high-level chatbot of all possible items which may or may not be relevant, 2. the log of the conversation between the user and the high-level LLM, 3. The response of the high-level chatbot which is a list of ingredients you will use as a guide to ensure you include all necessary ingredients and 4. the user profile which gives you 6 quantitative and 2 qualitative metrics to inform your judgement. Your job is to output in a friendly manner all the relevant ingredients from your context that satisfy the users request.

Firstly, the context generated by the high-level LLM is of the format: 'productName': '¡Name of the product¿', 'price': ¡Cost of the product¿, 'discount': ¡Type of discount¿ + ¡retrieved context¿

Secondly, the log of the conversation between the user and the high-level LLM - + ¡chat log¿

Thirdly, the list of items broken down by the high-level LLM that you must use as a guide to ensure you list all ingredients are - + ¡output of high-level LLM¿

Fourthly, the user profile is given as the user input below. If multiple items exist for a particular category, use these metrics to help you make your decision. The metrics are: price consciosuness where a higher value means they prefer cheaper products, brand loyalty where the user prefers branded items over non branded if the choice is there, helpAppreciation where a higher score means more hand holding and better longer responses, degreeKnowledge where a lower score indicates they know lesser about the items and products so will need more recommendations, speedShopping where they want to shop faster and make quicker decisions, newExplore where the user wants to try out new things if higher

Explain all decisions you make by reasoning it out. You need to output a nice formatted list of relevant items and as you give their details, location etc, also mention why you chose that item clearly.

All prices must be given in Euros and not USD. Also, ensure to keep the answers brief and to the point. If you dont, find the product, say 'Sorry, we dont have that.'

One final important point. Remember that your response is actually shown to the user. So present it as a nice list without telling them information they already know or gave you–just list the products neatly and explain your choices next to your selection so that when printed it is in a human readable format.

11.3 Low-Level LLM

The prompt presented below is used to instruct the low-level LLM to take the user query as input and output in a human-readable and friendly manner the retrieved item(s), modifcations to the previous list or answers to any other queries. The variables that are provided as input in this case are the retrieved context comprising of relevant items, the complete overall chatlog for conversation context, the user profile and user query added as the user input.

System Prompts:

You are a low-level assistant supermarket chatbot that handles more direct and specific queries of a customer. You take three inputs: 1. the current state of the conversation which tells you what items are currently recommended. This will be useful information for requests related to additions, substitutions or deletions. 2. the context–a list of retrieved items obtained by an information retrieval system after converting the user's query into an embedding and finding products that are close in vector space. 3. the user query–a question you have to answer in a friendly manner

Firstly, the current state of the conversation is provided here + ¡chat log¿

Secondly, the user's query was converted into embeddings and searched in the database to get some potential relevant results. This could be useful in case the user asked to add or substitute something from the original list. The 10 items obtained from the user query are - + ¡retrieved items as context¿

Lastly, the user's query will be provided below

Your job is simple–process the user query and based on the earlier interactions, context and nature of query, provide an updated response reflecting the necessary changes.

All prices must be given in Euros and not USD. Also, ensure to keep your responses friendly and helpful–you want to help the user as much as possible. If you cannot satisfy the user's query like adding or substituting a product outside the context provided above, say 'Sorry, we do not have that particular product' and provide potential alternatives based on the context if applicable and realistic.

ALWAYS INCLUDE ALL THE DETAILS OF THE PRODUCT SUCH AS NAME, PRICE, DISCOUNT IF ANY AND SHELF NUMBER IN YOUR LIST. ENSURE YOU GIVE THE COMPLETE UPDATED LIST AFTER EVERY MODIFICATION OR ADDITION.

11.4 Robot Destination Extractor LLM

The prompt presented below is used to instruct the final LLM to go over the last message of the chatbot and compile the list of destinations in a correct format.

System Prompts:

You are a LLM that takes the last message of a chatbot as input and outputs just a single line - 'Destinations - [¡comma separated list of shelfnumbers¿]'

Firstly, the last message of the chatbot is provided here -" + ¡last message of chatbot¿

The above message will have a lot of text along with some shelf numbers written directly as shelf numbers or shelf_no or equivalent.

You must read the whole message and output a single line saying : 'Destinations - [shelf¡number of first shelf¿,shelf¡number of second shelf¿,...]'

The format is absolutely essential. Here is a random example of how it should look like : 'Destinations - [shelf21,shelf36,shelf28,shelf1]' if the message above mentions these shelves.

If the same shelf number is present more than once, you must not add it after the first time. We are creating a set and every element in a set is unique.

STICK TO THE FORMAT CLOSELY AND ENSURE YOU OUTPUT ONLY THAT LINE.

11.5 Supermarket Data Creation LLM

The prompt presented below is used to instruct ChatGPT to help create the necessary data used in this study by creating the necessary code used to add items directly to the MongoDB database by writing an appropriate script in Python.

Prompt to ChatGPT

Your job is to help me update my MongoDB database in a particular manner. For this, you will augment data based on the requirements I provide you.

This is the current format of the collection - _id 100 shelf_no 1 productName ”Fresh Fruits”

You will write a Python program to modify this to an equivalent of - ”_id”: 100, ”shelf_no”: 1, ”categoryName”: ”Fresh Fruits”, ”products”: [”productName”: ”Apple 1kg”, ”price”: 1.73 , ”productName”: ”Orange 1kg”, ”price”: 1.60 , ”productName”: ”Banana 1kg”, ”price”: 1.89]

Do note that all prices are in Euros. And you need to create way more examples. The exact number is up to you. But you need to provide both product name, price and discount.

Make a list of all ’n’ products and write a python program to update it with this information. Each new prompt Ill specify the new category name and you will repeat the same for that category. Note: the number of items will very for each category. Some may be big others small. I need minimum 20 for each but more are always welcome. Try to be as exhaustive as possible.

For other categories like ’Chips’ or ’Cookies’ I need you to also say the brand name like Lay’s or Oreo in product name. Be as exhaustive and descriptive as possible.

Context: You need to augment the data based on the items seen in Dutch supermarkets like Jumbo, Albert Heijn, Lidl and Aldi. Think of the relevant ones and put it.

You may begin with fresh fruits

12 DESIGN AND CREATION OF THE STATE-OF-THE-ART GPT

The GPTs by OpenAI are the state-of-the-art AI agents that anyone can make without code. In this section, we will go over the design and implementation of the supermarket GPT which you can try on this link: https://chat.openai.com/g/g-A1zUUlrN6-supermarket-assistance-gpt

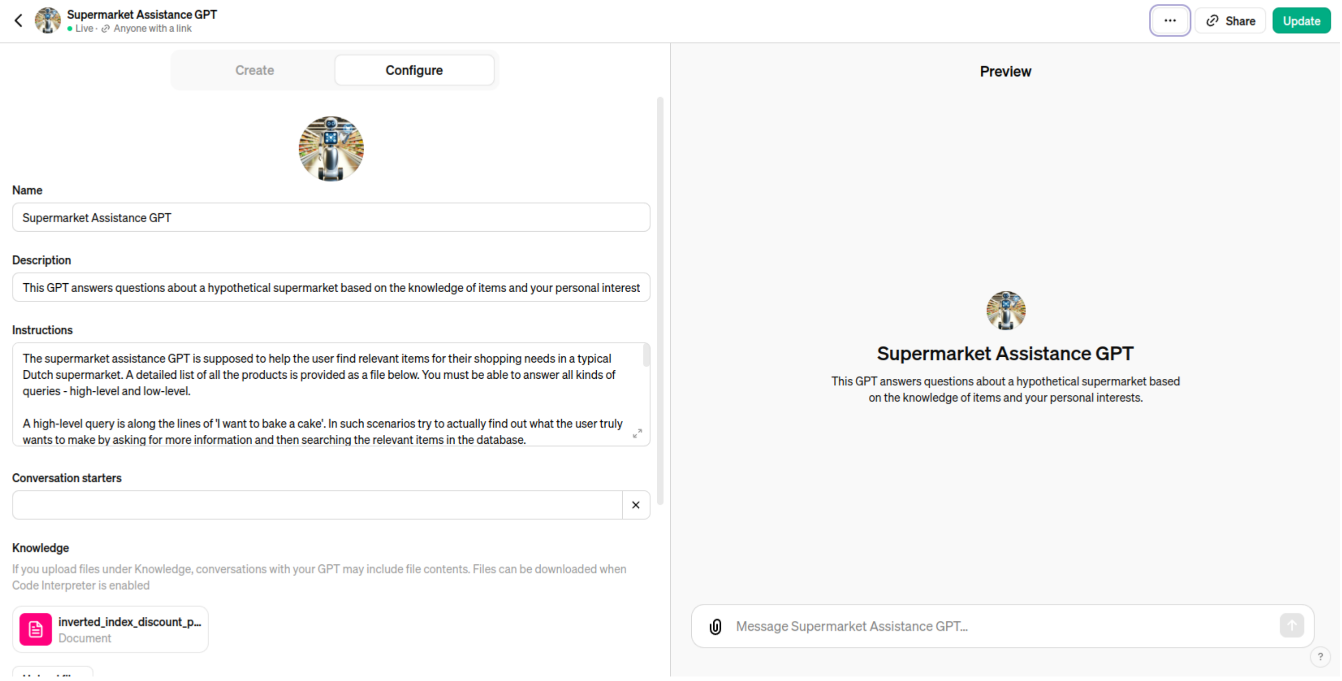

Figure S3. Layout of the GPT creation and customisation window

The GPT is provided with a comprehensive instruction to help it answer the necessary questions posed by a customer along with the inverted index database of all items for it to look up the relevant information. In the experiments performed, the user profile is manually entered, though in future iterations, it can be automatically retrieved via a function call to the database.

GPT Instruction:

The supermarket assistance GPT is supposed to help the user find relevant items for their shopping needs in a typical Dutch supermarket. A detailed list of all the products is provided as a file below. You must be able to answer all kinds of queries: high-level and low-level.

A high-level query is along the lines of 'I want to bake a cake'. In such scenarios try to actually find out what the user truly wants to make by asking for more information and then searching the relevant items in the database.

A low-level query is more direct and involves directly retrieving the relevant information from the data provided or making amendments to the previously displayed list shown to the customer.

Use only the data provided to you as your source of information. Disregard everything else. If something does not exist in the data provided say sorry we do not have that. All prices must be in Euros.

The user profile is given below including their name. If multiple items exist for a particular category, use these metrics to help you make your decision. All scores are from 1-5. The metrics are: price consciousness where a higher value means they prefer cheaper products, brand loyalty where the user prefers branded items over non-branded if the choice is there, helpAppreciation where a higher score means more hand holding and better longer responses, degreeKnowledge where a lower score indicates they know lesser about the items and products so will need more recommendations, speedShopping where they want to shop faster and make quicker decisions, newExplore where the user wants to try out new things if higher. There are also 2 qualitative questions namely dietaryPreferences and productInterest which is information written by the user.

¡User profile¿

Personalise your responses based on this. Respond in a friendly manner and feel free to use their name to talk to them like a helpful agent who wishes for the customer to get exactly what they are looking for.

The GPT was not provided access to other advanced capabilities such as Web Browsing, Code Interpreter and DALL·E Image Generation to level the playing field with our model and prevent the GPT from using other sources of information, which could potentially mislead it and lead to hallucinations. The inverted index was provided as a txt file that the GPT is capable of automatically chunking and storing.

Overall, creating the GPT was extremely simple and took an insignificant time to create and deploy compared to the custom multi-LLM agent discussed in the paper.

13 INFORMED CONSENT - CONVERSATIONAL AGENT EVALUATION EXPERIMENT

Research Study Invitation:

You are being invited to participate in a research study titled Evaluation of Conversational Agents for Shopping Market applications.

The purpose of this research study is to evaluate the conversational agent against the control to see if participants prefer one over another and measure any perceived benefits of their selected model and will take you approximately 20 minutes to complete. The data will be used for analysis and the information will be published post analysis in a research paper. We will be asking you to fill questionnaires and provide qualitative feedback on the experiences along with the possibility of storing the chatlogs without saving any personally identifiable information.

As with any online activity the risk of a breach is always possible. To the best of our ability your answers in this study will remain confidential. We will minimize any risks by making the study completely anonymous and no means of tracking the responses to the participant are present.

Your participation in this study is entirely voluntary and you can withdraw at any time. You are free to omit any questions. The data will be deleted after analysis and the completion of my thesis or latest by 31 st May 2024.

TEMPLATE 2: Explicit Consent points

Please make sure that you select (and amend as necessary) any Explicit Consent points which are relevant to your study and exclude those which do not apply. You should also add further points and necessary to address your specific research situation.

PLEASE TICK THE APPROPRIATE BOXES	Yes	No
**A: GENERAL AGREEMENT – RESEARCH GOALS, PARTICPANT TASKS AND VOLUNTARY PARTICIPATION**		
1. I have read and understood the study information dated 06/03/2024 or it has been read to me. I have been able to ask questions about the study and my questions have been answered to my satisfaction.	☐	☐
2. I consent voluntarily to be a participant in this study and understand that I can refuse to answer questions and I can withdraw from the study at any time, without having to give a reason.	☐	☐
3. I understand that taking part in the study involves answering online questionnaires, providing anonymous feedback about the different chatbots and realising the completely anonymous logs of your interactions may be saved for future model tuning	☐	☐
5. I understand that the study will end by the conclusion of my thesis defense and/or latest by May 31st 2024.		
**B: POTENTIAL RISKS OF PARTICIPATING (INCLUDING DATA PROTECTION)**		
6. I understand that taking part in the study involves the following risks of your anonmous responses being used for chatbot alignment and analysis of data to be published in a research paper. I understand that these will be mitigated by ensuring complete anonymity and not collecting any personally identifiable information	☐	☐
7. I understand that taking part in the study also involves collecting specific associated personally identifiable research data (PIRD) such as responses to the chatbot and/or your choices and entries in the survey with negligible risk of my identity being revealed since no PII is collected and everything is anonymous.	☐	☐
9. I understand that the following steps will be taken to minimise the threat of a data breach, and protect my identity in the event of such a breach - ensuring that no PII is collected and the questionnaires are completely anonymous	☐	☐
**C: RESEARCH PUBLICATION, DISSEMINATION AND APPLICATION**		
12. I understand that after the research study the de-identified information I provide will be used for publication for my thesis and potentially in a research paper.	☐	☐
13. I agree that my responses, views or other input can be quoted anonymously in research outputs	☐	☐
**D: (LONGTERM) DATA STORAGE, ACCESS AND REUSE**		
16. I give permission for the de-identified questionnaire and data logs that I provide to be archived in OneDrive in online only mode repository so it can be used for analysis up until the latest date when it will be destroyed.	☐	☐

14 DEMONSTRATION OF THE INTERACTION WITH THE MULTI-LLM CHATBOT

The video demonstration of the chatbot can be found by clicking the following link:

https://vimeo.com/932728461?share=copy

This section will cover the demonstration of the multi-LLM chatbot with the explanation of each step and how the agent works. The customer first begins by signing up to the supermarket S4 by adding their name, filling 6 Likert scale questions and 2 qualitative ones. These are then stored in the database and getting a QR code that serves as their membership card S5. The QR code encodes the member's unique ID which is used to identify them during future visits.

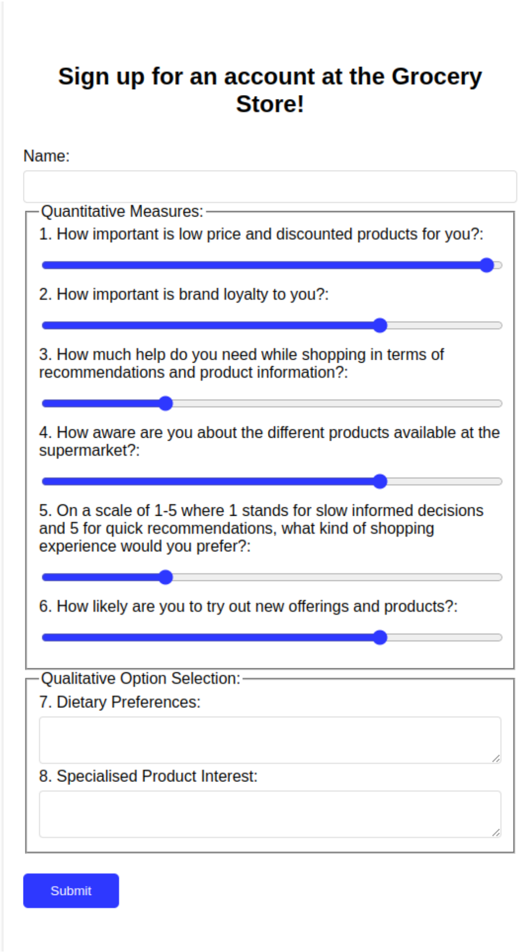

Figure S4. Signup window where the customer can fill in their preferences

Figure S6 shows the opening screen of the chatbot. It has been consciously designed to look similar to the GPTs by OpenAI. The agent also allows recording audio and using the text bar to provide the necessary information.

Figure S7 shows the high-level LLM at work. As we can see the query of asking for items for a protein smoothie is classified as a high-level request and the high-level LLM begins by asking user-specific questions, such as what protein powder they would prefer and what fruits would be ideal. The user continues by providing the necessary details and rough list of items is created.

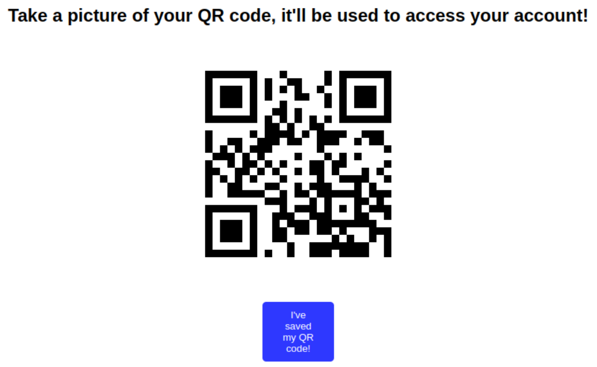

Figure S5. QR code that serves as a membership card for future visits and usage. This QR code is also used to retrieve the customer's profile

Once the user is satisfied with the basic list of items, Fig. S7 shows the mid-level LLM in action. It takes the list of all the basic items, searches for their availability and gives a list of relevant products with reasoning behind its choices. The user can then make changes to this list as seen in figure S9, where the customer asks for vanilla essence since the extract was unavailable and addition of Oreos. The low-level LLM is directly able to perform information retrieval and find the relevant information and details, such as their price and location. The updated list is provided afterwards as well.

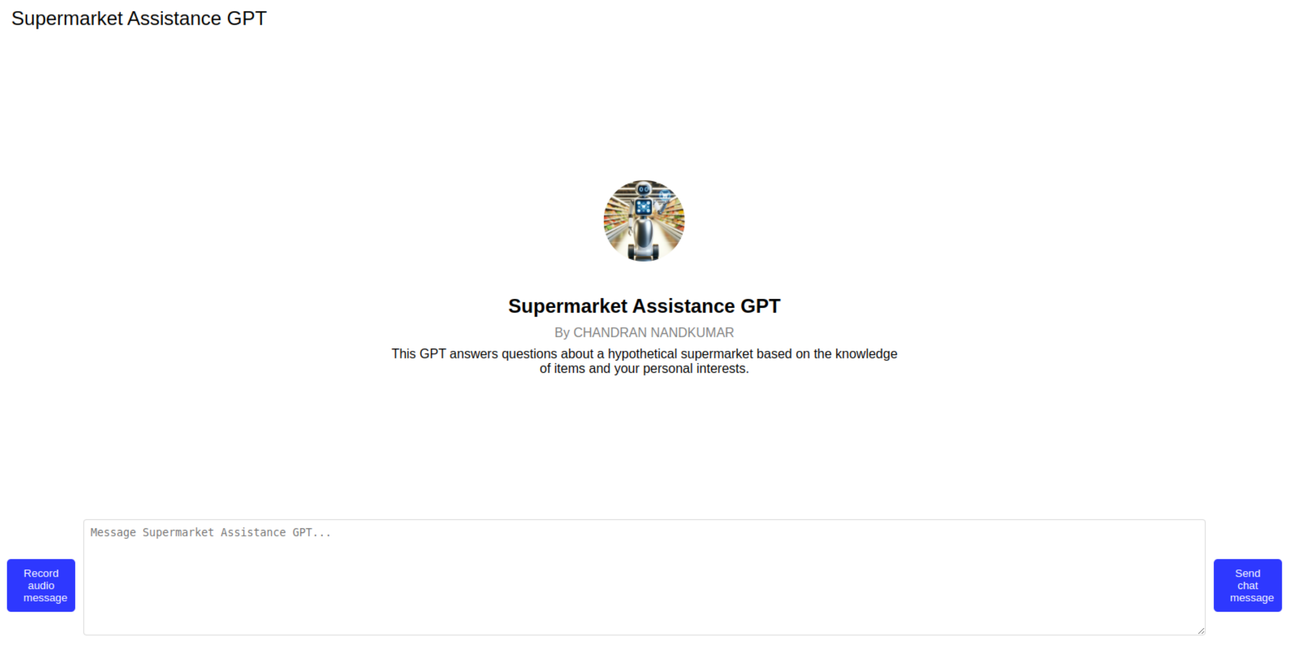

Figure S6. Opening window after scanning the QR code. The design is made similar to the GPT but with the addition of record audio option for voice-based interaction.

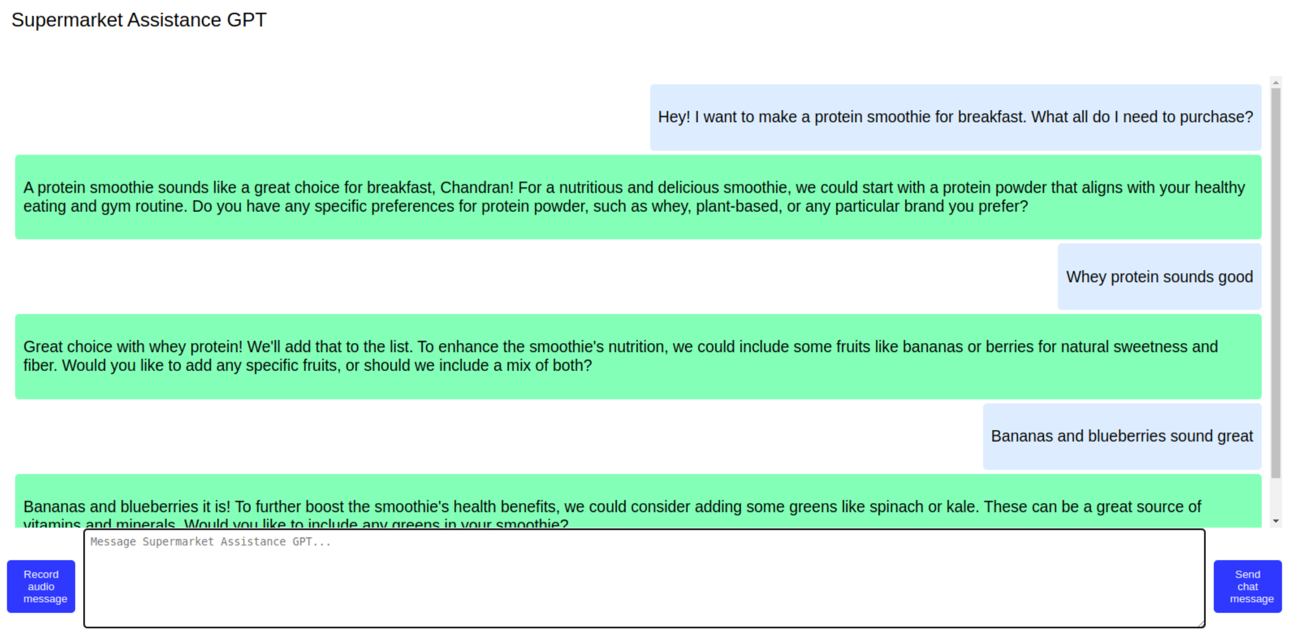

Figure S7. Here we see the high-level LLM at work. The high-level LLM asks for more questions and information based on the previous information collected about the user during signup (in this case, dietary preferences healthy and diverse diet.)

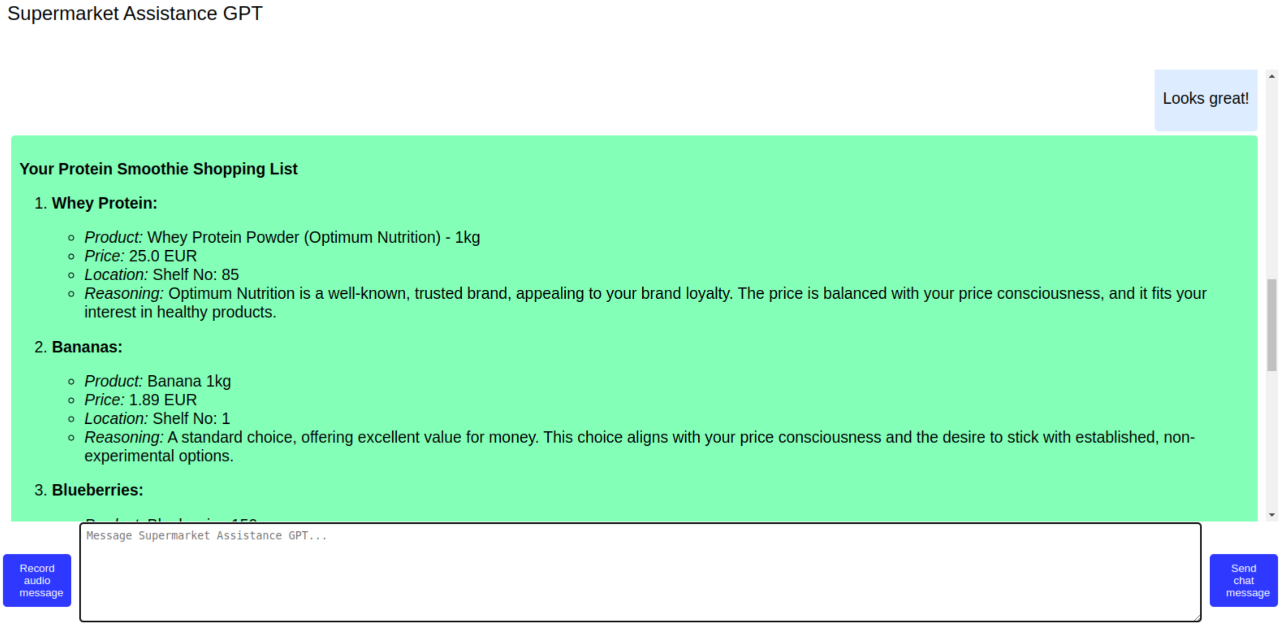

Figure S8. Here, after the user confirms they are happy with the list made by the high-level LLM, we see the medium-level LLM at work. It provides all the retrieved items, their price and availability.

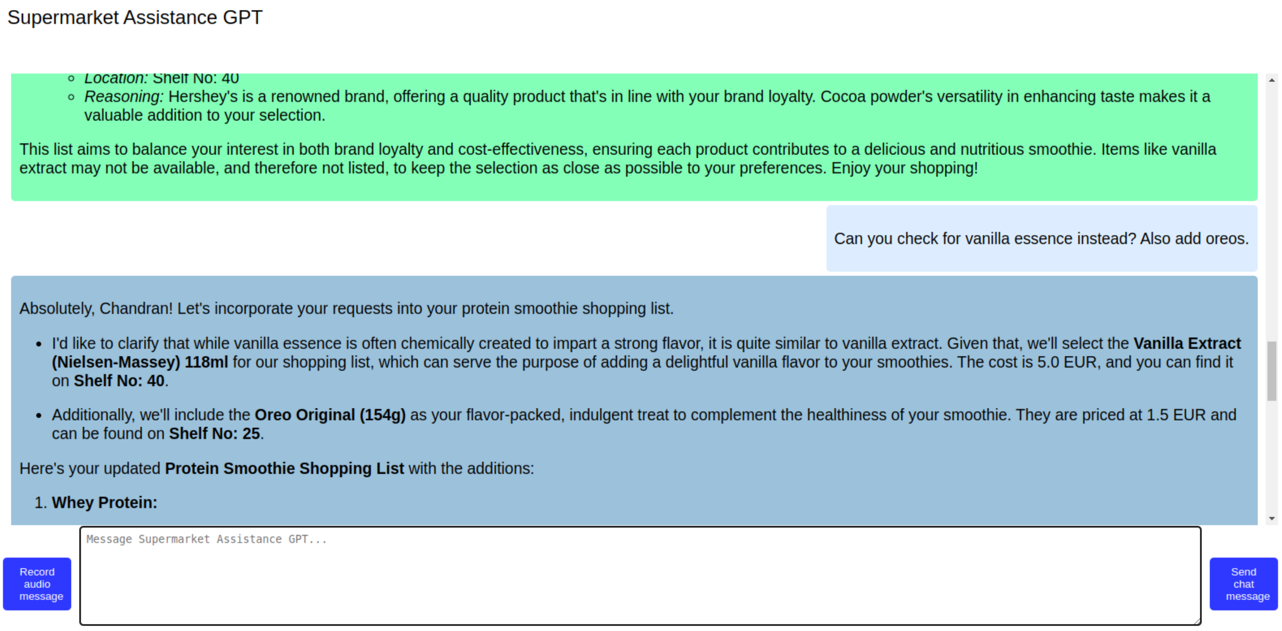

Figure S9. Here, the user wishes to make some modifications to the previously created list. The low-level LLM takes over and adds the necessary products to the list.
